# Intact but Protracted Facial and Prosodic Emotion Recognition Among Autistic Adults

**DOI:** 10.1007/s10803-025-06786-z

**Published:** 2025-03-27

**Authors:** Robert M. Jertberg, Sander Begeer, Hilde M. Geurts, Bhismadev Chakrabarti, Erik Van der Burg

**Affiliations:** 1 Section of Clinical Developmental Psychology, Vrije Universiteit Amsterdam and The Netherlands and Amsterdam Public Health Research Institute, Amsterdam, The Netherlands; 2 Dutch Autism and ADHD Research Center (d’Arc), Brain & Cognition, Department of Psychology, Universiteit van Amsterdam, Amsterdam, The Netherlands; 3 Leo Kannerhuis (Youz/Parnassiagroup), Amsterdam, The Netherlands; 4 Centre for Autism, School of Psychology and Clinical Language Sciences, University of Reading, Reading, UK; 5 India Autism Center, Kolkata, India; 6 Department of Psychology, Ashoka University, Sonipat, India

**Keywords:** Autism, Emotion recognition, Facial expressions, Affective prosody

## Abstract

Despite extensive research efforts, it is unclear how autistic and non-autistic individuals compare in their ability to recognize emotions. Differences in demographic and task factors have been proposed as explanations for divergent findings, but limitations in samples and designs have obscured insight into this possibility. This study investigated the extent of emotion recognition differences among autistic adults and the influence of these factors upon them. We recruited a large sample of autistic and non-autistic adults (*N* = 1,239) spanning across adulthood (18–76 years). In three online experiments, we compared their performance in recognizing emotions from basic facial expressions, complex expressions conveyed by the eyes alone, and prosodic elements of speech. Autistic individuals performed as well as non-autistic ones in terms of recognition accuracy/sensitivity across measures and emotional categories but took longer to do so. We also detected comparable influences of age, estimated intelligence quotient, and gender (as well as task demands) on both groups. While autistic adults may differ in how they process emotional stimuli, they can do so effectively when given sufficient time. Accordingly, efforts to help autistic individuals improve their ability to recognize emotions may be more fruitful if they focus on efficiency over accuracy. Additionally, reaction time data may offer greater insight than accuracy into differences between autistic and non-autistic individuals on emotion recognition tasks. The similar effects of the demographic and task factors we analyzed on both groups suggest that explanations of the discrepancies in prior literature lie elsewhere.

**Supplementary Information:**

The online version contains supplementary material available at 10.1007/s10803-025-06786-z.

Research suggests that autistic people^1^ may show impairments in the ability to infer others’ emotional states from perceptual cues (Harms et al., 2010; Leung et al., 2022; Lozier et al., 2014; Uljarevic & Hamilton, 2013; Williams et al., in press; Yeung, 2022; Zhang et al., 2022). Emotion recognition has clear relevance to social reciprocity and nonverbal communication behavior, differences in which are diagnostic criteria of autism (American Psychiatric Association, 2013). Additionally, across studies with autistic participants, a link between emotion recognition abilities in the lab and everyday social functioning has been established (Trevisan & Birmingham, 2016). As such, these impairments may be a key feature of autism with considerable diagnostic importance. However, while there are clear trends in the literature, differences in demographic factors and task design have contributed to mixed findings, especially among autistic adults. In this study, we seek to elucidate the influence of these factors by measuring facial and prosodic emotion recognition in a large cohort of autistic and non-autistic adults with novel analyses investigating the roles of age, stimulus complexity, and time, as well as the relationships between these different aspects of emotion recognition.

Facial expressions and the sounds of speech are two of the most salient and socially relevant types of emotional cues, and the former is the most widely studied topic of emotion recognition in autism research (Uljarevic & Hamilton, 2013). Although findings comparing the performance of autistic and non-autistic individuals have been quite mixed (Harms et al., 2010), meta-analyses have found significant evidence of impairment of facial emotion recognition in autism (Lozier et al., 2014; Uljarevic & Hamilton, 2013; Yeung, 2022). Recognition of vocal emotions is a newer, emerging field. Changes in pitch, intonation, and speed are able to convey emotional meaning that goes beyond the semantics of spoken words, a linguistic feature known as affective prosody (Frick, 1985). Due to the overlapping differences in emotion recognition and speech processing in autism (Georgiou & Spanoudis, 2021; Kwok et al., 2015; Leung et al., 2022; Rump et al., 2009), one might expect affective prosody recognition to be especially impaired. However, the only meta-analysis on the topic found that the group difference initially detected was no longer significant after accounting for publication bias, highlighting the necessity for more research with larger samples (Zhang et al., 2022). What, then, contributes to these inconsistencies in findings?

Differences in demographic factors are one possible source of variation in outcomes, particularly given that the field often includes small samples with skewed distributions. For example, the vast majority of studies are heavily biased towards male participants, making it difficult to ascertain the importance of gender to potential differences between groups. Women tend to perform better on facial and prosodic emotion recognition tasks generally (Fujisawa & Shinohara, 2011; Greenberg et al., 2023; Hall, 1978; Lausen & Schacht, 2018), and some studies suggest that autistic women may not encounter the same degree of difficulties seen among autistic men in these areas (Ketelaars et al., 2016; Kothari et al., 2013; Sucksmith et al., 2013). This underscores the need for larger, more balanced samples when drawing conclusions about autism across genders.

As autism is a developmental condition, the most widely investigated demographic factor at play is age. Among non-autistic individuals, both facial and prosodic emotion recognition develop gradually throughout childhood, reaching maturity during adolescence (Filippa et al., 2022; Herba & Phillips, 2004; Lawrence et al., 2015). Autism has been theorized to involve disruption of this typical maturation trajectory (Harms et al., 2010; Lozier et al., 2014; Rump et al., 2009), which may translate into larger differences between autistic and non-autistic adults than children. Conversely, individuals with autism may develop compensatory strategies with age, reducing observable differences in emotion recognition (Harms et al., 2010; Livingston & Happé, 2017). These contrasting predictions have been met with equally contrasting findings. Some studies and meta-analyses have shown greater differences between autistic and non-autistic adults than children in facial and prosodic emotion recognition (Leung et al., 2022; Lozier et al., 2014; Rump et al., 2009), whereas others have not detected an influence of age (Uljarevic & Hamilton, 2013; Yeung, 2022; Zhang et al., 2022). Additionally, facial and prosodic emotion recognition abilities have been found to deteriorate at advanced ages among the general population (Cortes et al., 2021; Lambrecht et al., 2012; Ruffman et al., 2008, 2009). However, no studies to date have investigated differences between autistic and non-autistic individuals in the age range at which this occurs, so the degree to which emotion recognition may differ in autistic adults across the lifespan is unclear.

Divergent findings with regard to age may also have to do with shifting demands between more challenging tasks used with adults and simpler ones used with children (Harms et al., 2010). Some research suggests that differences between autistic and non-autistic individuals are more pronounced with complex stimuli from emotional categories that develop later (Leung et al., 2022; Lozier et al., 2014; Uljarevic & Hamilton, 2013). As such, studies that use only a small range of simple stimuli may be unable to detect differences between groups that studies with more complex stimulus sets covering more emotional categories can.

Another crucial factor may be processing speed. Some studies have found that autistic adults and children are just as accurate as non-autistic ones in recognizing facial and prosodic emotions, but that they are simply slower in doing so (Leung et al., 2023; Matsumoto et al., 2016). This resonates with findings that autistic individuals may use compensatory mechanisms to augment their performance (Harms et al., 2010) and do better on the featural than holistic level (Yeung, 2022) in emotion recognition. Both of these factors could translate into a longer time taken to reach optimal performance. They might also require a greater degree of effort, leading to a deterioration in performance over the course of long experiments. However, because most studies do not measure reaction times (RTs) or performance over the course of trials, it is difficult to determine the extent to which temporal factors may be relevant.

Throughout the literature, overlapping potential confounds obfuscate the influence of the various factors that may explain discrepancies in facial and prosodic emotion research. Beyond the potential diagnostic utility of measuring these abilities, given that emotion recognition training has been shown to be effective for autistic adults and children (Baron-Cohen et al., 2009; Farashi et al., 2022; Zhang et al., 2022), identifying those who struggle in these areas could allow targeted interventions to improve related social skills. As such, it is crucial that we better understand the influence of underlying demographic factors and design features on the pattern of findings thus far. To this end, we recruited a large sample of autistic and non-autistic adults with better representation of autistic women than in most earlier research as well as wide ranges of ages. We administered the most popular facial emotion recognition paradigms- (a) the Karolinska Directed Emotional Faces (KDEF, Lundqvist & Öhman, 1998; Sucksmith et al., 2013; Wilson et al., 2014), (b) the Reading the Mind in the Eyes Test (RMET, Baron-Cohen et al., 2001)- and (c) an affective prosody paradigm allowing fine grained insight into new features of vocal emotion recognition (Van der Burg et al., 2024). Together, these paradigms allowed us to investigate: (a) whether autistic and non-autistic adults differ in facial and/or prosodic emotion recognition speed or accuracy, (b) which demographic (i.e., age, gender, and IQ) and task (i.e., stimulus type/complexity) variables might influence performance, (c) how performance might differ between groups over the course of the experiment, (d) how strongly our different measures correlate, and (e) which task is most predictive of a clinical diagnosis.

## Methods

*Participants.* The three experiments described in this paper were part of a larger two-part battery. Overall, a total of 706 autistic participants were recruited via the Netherlands Autism Register (NAR, https://nar.vu.nl/), and 533 non-autistic participants were recruited via the NAR as well as Prolific Academic, an online recruitment platform that compares favorably to popular alternatives in data quality (see Peer et al., 2022). The autistic participants reported a formal diagnosis by a qualified, independent clinician. The non-autistic participants reported no diagnosis of autism. NAR participants received €15 gift cards and Prolific Academic participants were paid £15. All participants were fluent in Dutch, naïve to the purpose of the study, and gave informed consent prior to the experiment. Dropout and exclusion rates for individual measures differed according to which experiments came earlier in the batteries, data availability for participants, and outlier criteria pre-registered in As Predicted (#148205, https://aspredicted.org/BZG_S1W).

Note that for Experiments 1 and 2, in addition to the pre-registered exclusion criteria, we considered a trial an outlier if the RT was < 500 ms (rather than the pre-registered 250 ms threshold). We decided our threshold was not strict enough after noticing that some participants had unrealistic mean response times close to 400 ms (with the mean being over 3000 ms in Experiment 1 and 6000 ms in Experiment 2). Furthermore, for Experiment 2, one participant was excluded from further analyses as (s)he opened another application (like email, facebook, etc.) on 85% of the trials (group mean < 1%). With regard to Experiment 3, we removed our minimum RT threshold (originally 250 ms) due to the fact that participants were able to prepare to respond while stimuli were playing. This led to a large number of very quick responses (16% < 250 ms). Detailed information on the exclusion process can be found in Supplementary Figs. 1–3. The demographic information for each experiment is depicted in Table 1.

**Table 1:** Demographic information for each experiment. Standard deviations are shown between parentheses. Asterisks signify significant group differences (*p* <.05)

	Experiment 1: KDEF		Experiment 2: RMET		Experiment 3: Prosody	
	Autism	No Autism	Autism	No Autism	Autism	No Autism
	(*n* = 399)	(*n* = 370)	(*n* = 516)	(*n* = 393)	(*n* = 398)	(*n* = 390)
Gender*						
Women (%)	66.4	49.2	64.3	51.7	65.8	48.5
Men (%)	33.6	50.8	35.7	48.3	34.2	51.5
Age*	44.7 (14.2)	32.4 (12.3)	44.9 (14.0)	33 (12.7)	44.9 (14.1)	32.1 (12.2)
Age Range	18–76	18–72	18–77	18–74	18–76	18–74
AQ-28*	83.4 (10.8)	60.5 (10.9)	82.8 (10.8)	59.9 (11.3)	83.5 (10.8)	60.6 (11.0)
AQ-28 Range	46–109	30–96	46–109	30–96	46–109	30–96
ICAR	0.53 (0.21)	0.52 (0.19)	0.53 (0.21)	0.53 (0.19)	0.53 (0.21)	0.52 (0.19)
ICAR Range	0-0.94	0.0-0.94	0-0.94	0.06–0.94	0-0.94	0-0.94

AQ: Autism Quotient

ICAR: International Cognitive Ability Resource (abbreviated intelligence quotient test)^a^

^a^The ICAR project is an open resource for online cognitive tasks. We used the ICAR-16 intelligence test, which correlates strongly with full-scale IQ (Young & Keith, 2020) and has been shown to be age and sex invariant (Young et al., 2019). We report proportions of correct responses, which we use as covariates in subsequent analyses

The experiments were approved by the ethical committee from the Vrije Universiteit Amsterdam (VCWE-2020-041R1) in accordance with the Netherlands Code of Conduct for Research Integrity. All experiments were programmed and conducted online using Neurotask (www.neurotask.com).

## Experiment 1: Basic Facial Emotion Recognition (KDEF)

### Stimuli

**Fig. 1:**
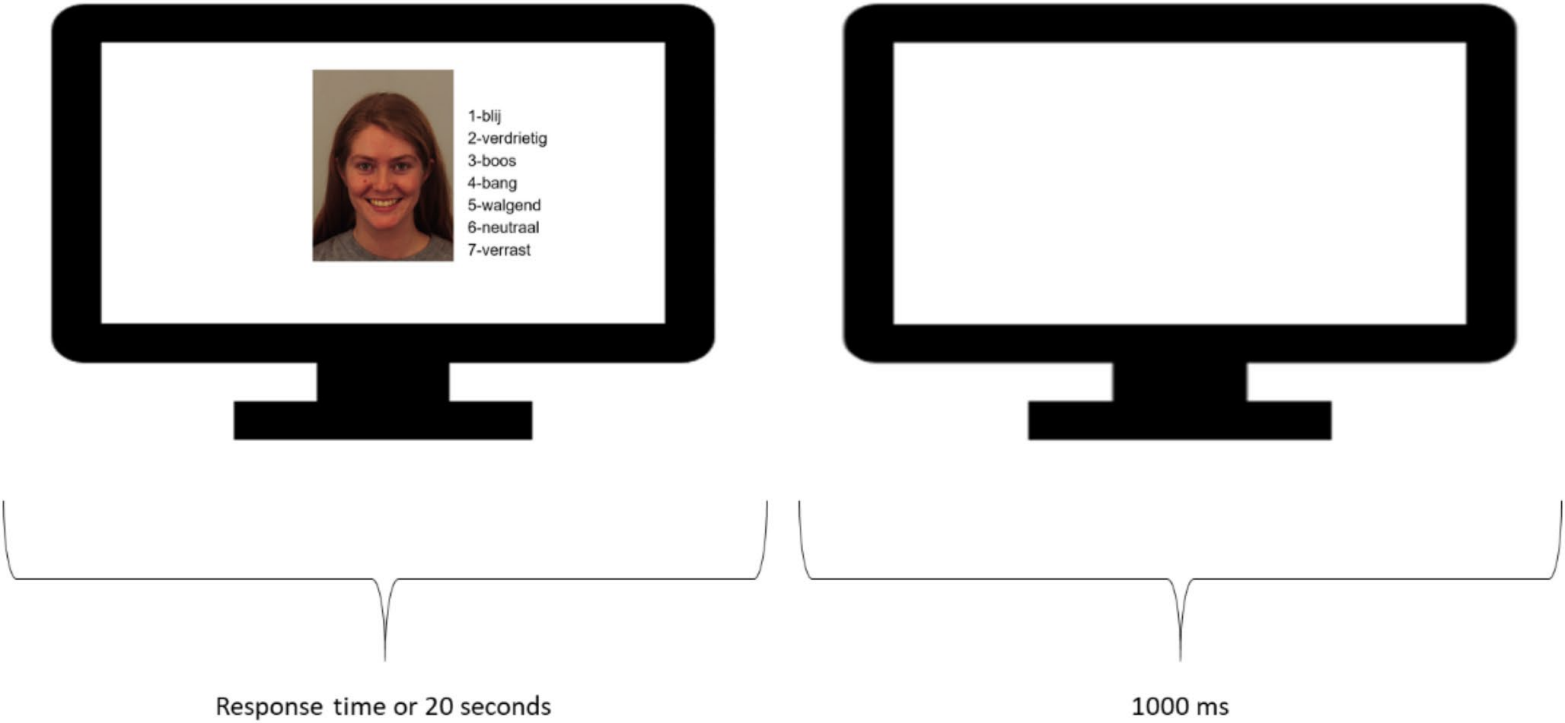
Example trial sequence from the Karolinska Directed Emotional Faces task (Lundqvist & Öhman, 1998). The monitor provides a proportionate representation of the dimensions/alignment of the images

Ten front-facing color photographs of actors/actresses making expressions from each of seven emotional categories (happy, sad, angry, afraid, disgusted, surprised, and neutral) were selected from the subset of the KDEF database used in Sucksmith et al. (2013). The images (562 × 762 pixels) were displayed centrally, shifted up 10% and taking up 50% of the available vertical screen space within the web browser (see Fig. 1 for an illustration). Words referring to each of the seven emotional classes were translated into Dutch (blij (happy), verdrietig (sad), boos (angry), bang (afraid), walgend (disgusted), neutraal (neutral), verrast (surprised) and displayed in a fixed order to the right of the image with corresponding numbers. The order was fixed to avoid participants confusing the numbers associated with specific terms. Psychometric properties of the stimulus set are explored in Goeleven et al. (2008).

### Procedure:

Figure 1 provides an example trial sequence.

Each trial, an image would appear along with the 7 word/number pairs. Participants were instructed to press the number key corresponding to the emotion they thought the person in the image was experiencing as quickly and accurately as possible. They were notified that they would have a maximum of 20 s per image to make their decisions. After a response was given (or 20 s elapsed, as in Sucksmith et al., 2013), a blank screen was shown for 1 s (to prevent participants skipping past two trials), and the subsequent trial was initiated. In total, there were 70 experimental trials (10 of each emotional category) appearing in randomized order. Prior to this experiment (as well as Experiments 2 and 3), participants received written instructions on the screen. There were no practice trials.

## Experiment 2: Complex Facial Emotion Recognition (RMET)

**Fig. 2:**
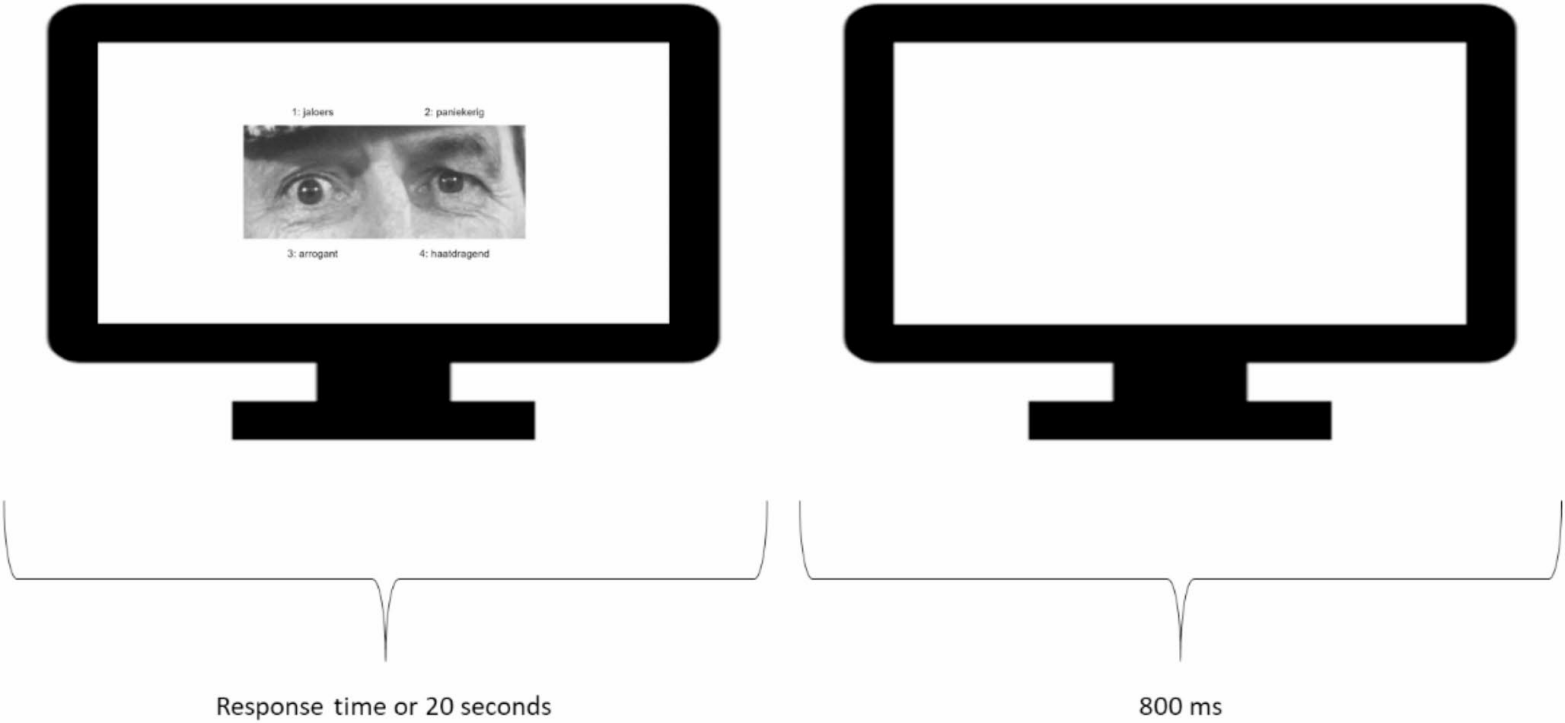
Example trial sequence from the reading the mind in the eyes task. The monitor provides a proportionate representation of the dimensions/alignment of the images

*Stimuli.* 37 black and white photographs of the eyes and surrounding area of actors/actresses making various emotional expressions were taken from Baron-Cohen et al. (2001). The images (approximately 550 × 225 pixels) were displayed centrally, taking up 100% of the available horizontal screen space (note: available screen width was equal to available screen height within the browser, see Fig. 2 for an example). Four words describing feelings (one of which corresponded to each expression) were displayed (along with numbers) equidistantly from the center of each image, towards the corners of the screen. The words were Dutch translations of those presented in the original study, taken from the EU AIMS project (see https://www.aims-2-trials.eu/about-aims-2-trials/eu-aims/). A different set of four words appeared along with each image, although some words repeated. Psychometric properties of the stimulus set are explored in Olderbak et al. (2015)

### Procedure:

Figure 2 provides an example trial sequence.

Each trial, an image would appear along with the four word/number pairs. Participants were instructed to read all four words, then press the number key corresponding to the option they felt best described what the person in the image was feeling as quickly and accurately as possible. They were notified that they would have a maximum of 20 s per image to make their decision. After a response was given (or 20 s elapsed), a blank screen was shown for 800 ms, and the next trial was initiated. Participants completed one practice trial and then 36 experimental trials in a fixed order (as in Baron-Cohen et al., 2001).

## Experiment 3: Affective Prosody Recognition

*Stimuli.* Four auditory tokens of the semantically neutral Dutch sentence, “zijn vriendin kwam met het vliegtuig (his girlfriend arrived by plane),” were taken from the stimulus set described in de Gelder and Vroomen (2000). The fundamental frequency, accented syllables, and duration of the stimuli were manipulated by the original authors to create a 7-step affective prosodic continuum from happy to fearful. Stimulus duration ranged from 1513 ms to 1755 ms (Van der Burg et al., 2024). We selected two stimuli from either side of the continuum (specifically the first, third, fifth, and seventh, with the first being the happiest, and the seventh being the most fearful) for our experiment. Psychometric properties of the stimulus set have not been explored extensively.

**Fig. 3:**
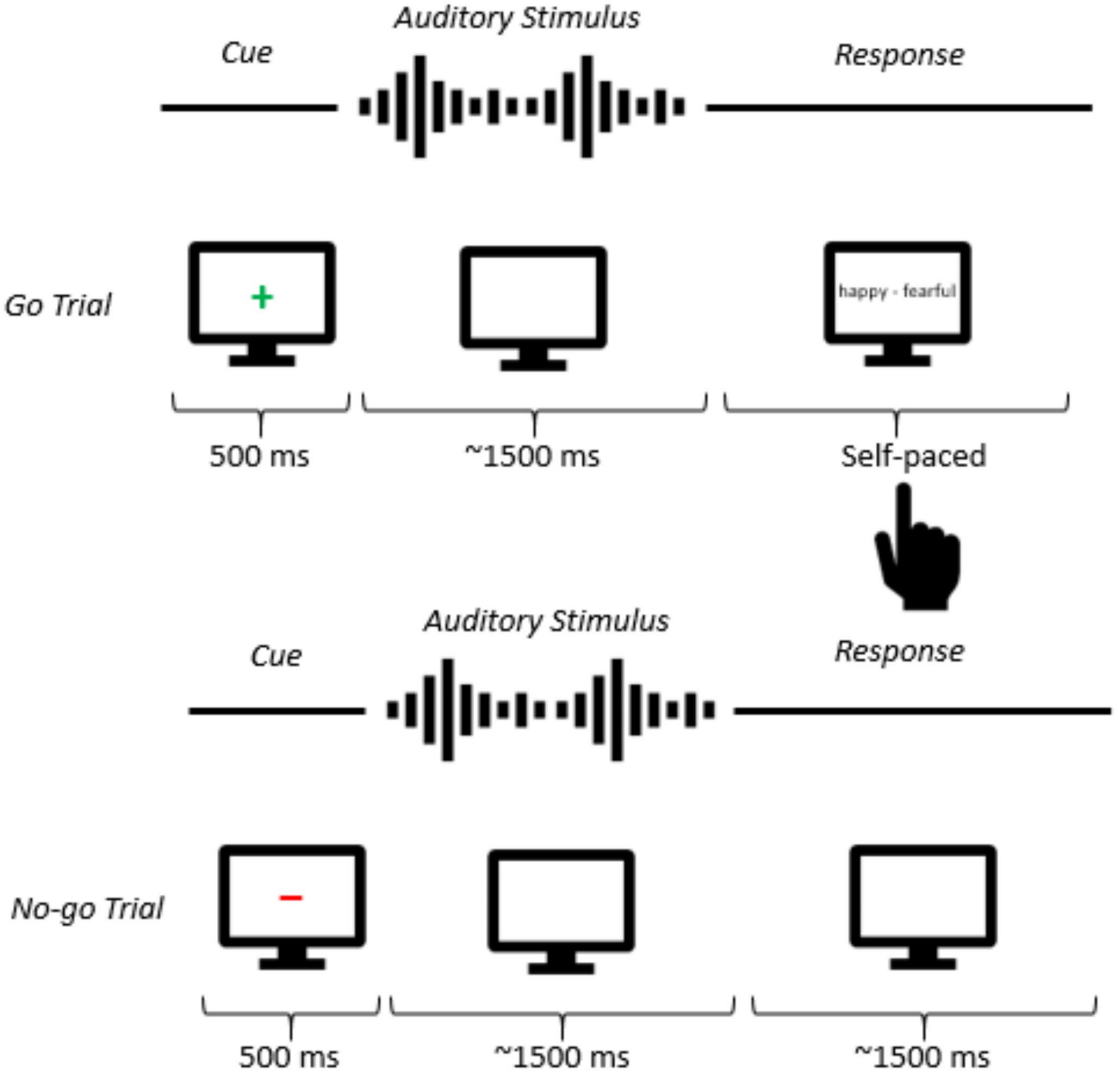
Example trial sequences from the affective prosody experiment. On every trial, participants saw a green + sign or red – sign, indicating the trial type (i.e., a go or no-go trial, respectively). Subsequently, an auditory stimulus was presented. In go trials, participants rated the emotional content of the stimulus (on a 7-point Likert scale). On no-go trials, participants were instructed to withhold their response

### Procedure:

Figure 3 provides example trial sequences.

Trials began with the presentation of a fixation symbol in the center of the screen for 500 ms. The symbol could be either a green + (indicating a “go” trial) or a red - (indicating a “no-go” trial)^2^. The fixation symbol then disappeared (leaving the screen blank) and the auditory stimulus was presented. Subsequently, on go trials, participants were instructed to respond to what emotion the voice expressed on a Likert scale of 1–7 (with 1 being happiest and 7 being most fearful) by pressing the corresponding number key (see also Van der Burg et al., 2024). A Likert scale was chosen because stimuli lied on a continuum between happy and afraid, rather than coming from specific categories (as in the previous experiments). The scale was displayed on the screen to help the participants. On no-go trials, participants were instructed to withhold their response (no response was recorded), and a blank screen was displayed for 3 s minus the stimulus duration. The subsequent trial was initiated when participants made a response (on go trials) or when the 3 s elapsed (on no-go trials). Time-outs were not implemented on go trials to remain consistent with Van der Burg et al. (2024). Participants completed 6 practice trials to get familiar with the task and stimuli used. Then, each of the four stimuli were presented 32 times for go trials, and the happiest and most fearful stimuli were presented 32 times for no-go trials. In total, this amounted to 192 experimental trials, presented in random order.

## Results

Alpha was set to 0.05, but Bonferroni corrected for multiple comparisons according to the number of dependent variables measured for each experiment. Because each experiment involved three dependent variables, this resulted in an alpha of 0.017 within measures. Statistical tests were conducted using Just Another Statistical Program (JASP, version 0.17.3.0, see Love et al., 2019) and Jamovi (version 2.3.28, see The Jamovi Project, 2024). The frequentist analyses on accuracy/sensitivity and RT reported in the main text were repeated using JASP’s Bayesian statistics module. The results of these analyses are shown in the Supplementary Tables 1–6.

## Experiment 1: Basic Facial Emotion Recognition (KDEF)

**Fig. 4:**
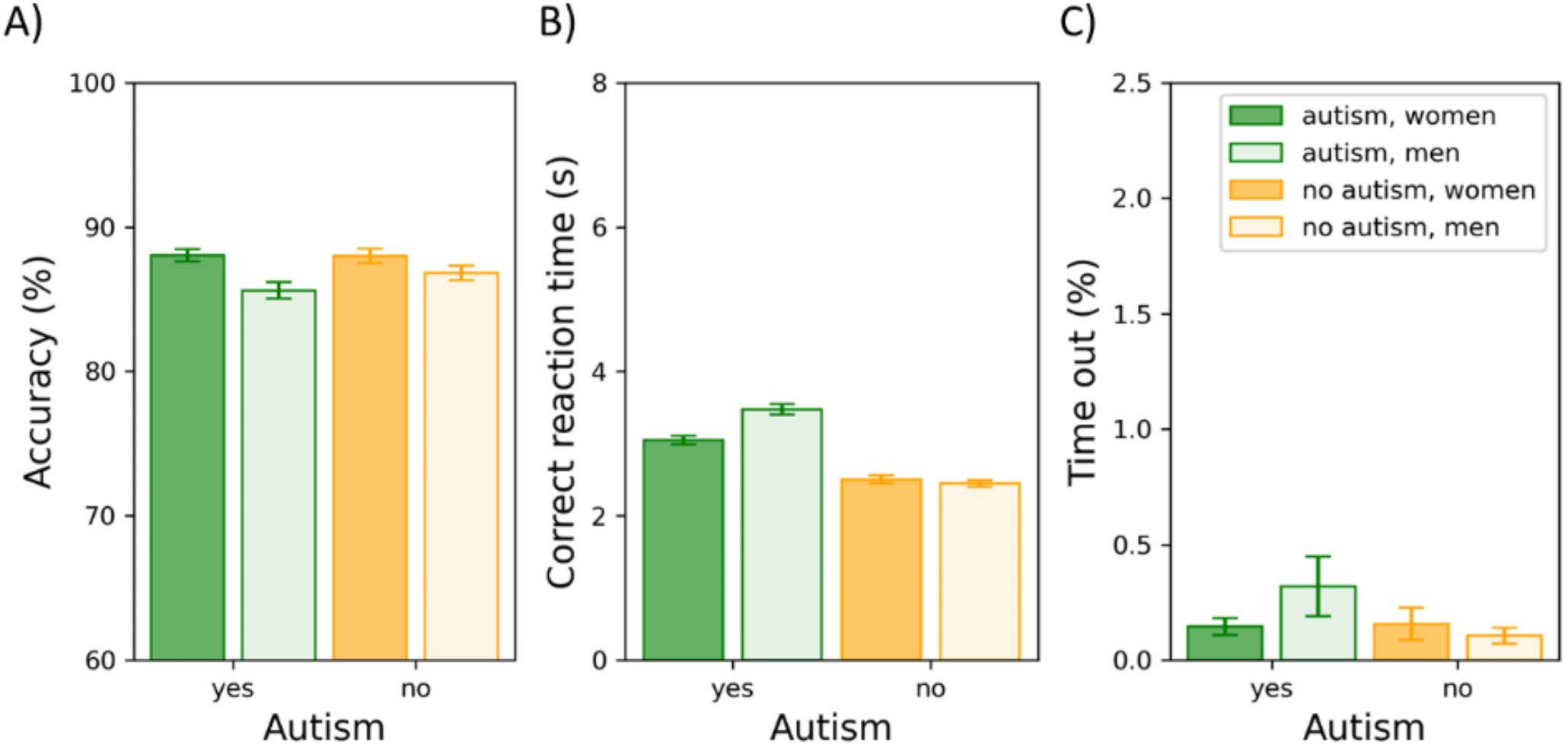
**A**) Mean accuracy as a function of group and gender. **B**) Mean correct reaction time as a function of group and gender. **C**) Mean percentage of time-outs as a function of group and gender. The error bars reflect the standard error of the mean

Figure 4 illustrates the mean accuracy (Panel A), mean correct RT (Panel B), and percentage of time-outs (Panel C) as functions of group for men and women. We conducted ANCOVAs on the mean accuracy, mean correct RT, and mean percentage of time-outs with group and gender as between-subject variables and age and ICAR scores as covariates.

*Accuracy.* The effect of group was not significant (*F*(1, 763) = 0.015, *p* =.903, η_p_^2^ = 1.961 × 10^− 5^). However, there was a significant main effect of gender (*F*(1, 763) = 9.854, *p* =.002, η_p_^2^ = 0.013), such that women (mean: 88%) performed better than men (86%). Age (*F*(1, 763) = 7.104, *p* =.008, η_p_^2^ = 0.009) had a negative relationship with accuracy (i.e., older people were less accurate), and ICAR score (*F*(1, 763) = 10.870, *p* =.001, η_p_^2^ = 0.014) had a positive one (i.e. those with higher scores performed better). All other *F* ≤ 0.308; all other *p* ≥.579.

*Reaction time.* There was a significant effect of group (*F*(1, 763) = 51.634, *p* <.001, η_p_^2^ = 0.063), such that autistic participants (3191 ms) were slower than non-autistic participants (2475 ms). Age also had a positive relationship with RT (*F*(1, 763) = 154.954, *p* <.001, η_p_^2^ = 0.169), i.e., older participant were slower. All other *F* ≤ 5.048; all other *p* ≥.025.

*Percentage time-out.* There were no significant effects on time-out rates (all *F* ≤ 2.928; all *p* ≥.087), which were extremely rare (0.02% of trials).

**Fig. 5:**
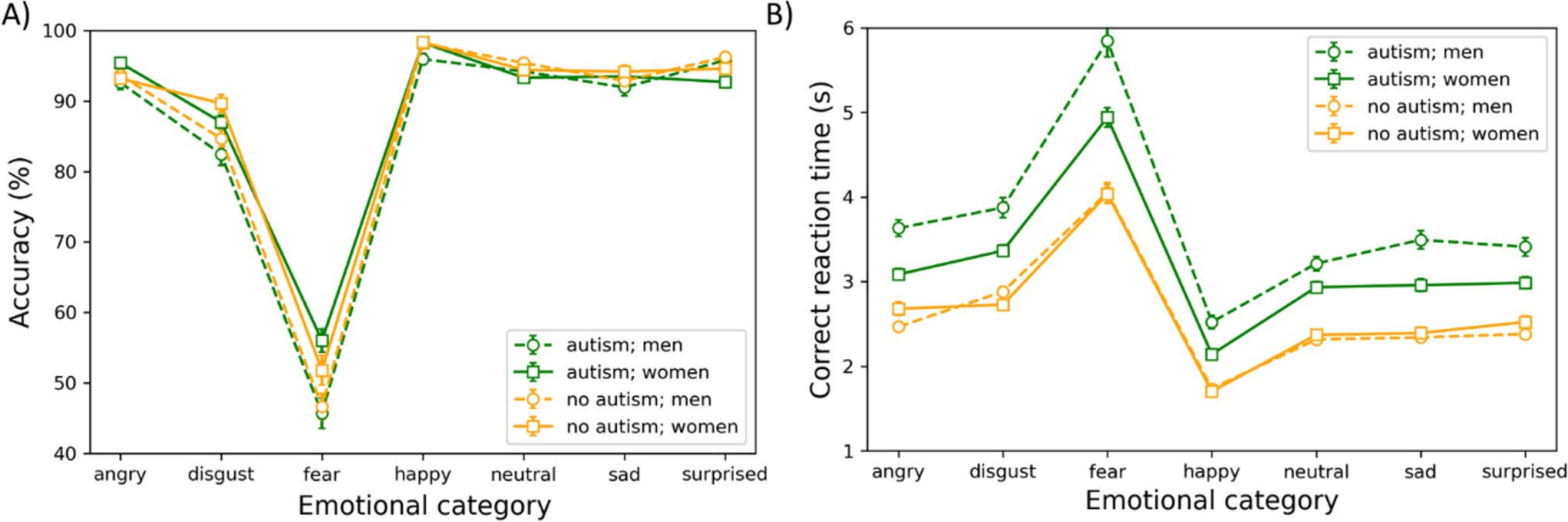
**A**) Mean accuracy as a function of the emotional category of the stimulus according to group and gender. **B**) Mean correct reaction time as a function of the emotional category of the stimulus according to group and gender. In both panels, the error bars reflect the standard error of the mean

Figure 5a and b reflect the mean accuracy and mean RT as functions of emotional category for each group and gender, respectively. To determine whether the groups differed on specific emotional categories (particularly those that were harder), we conducted exploratory mixed measures ANOVAs on the mean accuracy and mean correct RT with emotional category as a within-subject variable, group and gender as between-subjects factors, and age and ICAR scores as covariates.

*Accuracy by emotional category.* As in the main analysis, there was no main effect of group (*F*(1, 763) = 3.172 × 10^− 5^, *p* =.974, η_p_^2^ = 1.354 × 10^− 6^), but there were significant main effects of gender, ICAR, and age (all *F* ≥ 7.329; all *p* ≤.007). Emotional category had a significant main effect (*F*(6, 4578) = 101.244, *p* <.001, η_p_^2^ = 0.117), with accuracy ranging from 50.9% for fearful stimuli to 97.8% for happy stimuli, but it did not interact with group (*F*(6, 4578) = 0.793, *p* =.575, η_p_^2^ = 0.001). There were also significant interactions of emotional category with gender (*F*(1, 763) = 10.194, *p* <.001, η_p_^2^ = 0.013) and ICAR score (*F*(1, 763) = 4.183, *p* <.001, η_p_^2^ = 0.005). As our focus was on group differences, these interactions were not further explored. All other *F* ≤ 2.559; all other *p* ≥.018.

*Reaction time by emotional category.* As in the main analysis, the ANOVA yielded significant main effects of age and group (both *p* values < 0.001). The main effect of gender, which did not survive Bonferroni correction in the main ANCOVA (*p* =.025), was significant in the repeated measures ANOVA (*F*(1, 727^3^) = 9.357, *p* =.002, η_p_^2^ = 0.013), with women (2968 ms) responding more quickly than men (3066 ms). The main effect of category was significant (*F*(6, 4362) = 22.052, *p* <.001, η_p_^2^ = 0.029), with mean RTs ranging from 4669 ms for fearful stimuli to 2003 ms for happy stimuli. Category also interacted with all other factors (all *F* ≥ 2.809; all *p ≤*.010), including group (*F*(6, 4362) = 7.933, *p* <.001, η_p_^2^ = 0.011). The group × category interaction was further examined using two-tailed *t*-tests. The *t*-tests revealed a significant group difference for all categories (all *t* ≥ 8.653; all *p <*.001), suggesting that the group × category interaction reflects magnitude differences across them. Cohen’s *d* ranged from 0.625 for surprised faces to 0.745 for neutral faces (see Fig. 5b for a graphical representation of these differences). The three-way interaction of group, gender, and category did not reach significance after Bonferroni correction (*F*(6, 4362) = 2.304, *p* =.032, η_p_^2^ = 0.003). The interactions of category and the other factors were not investigated further as our focus was on group differences.

**Fig. 6:**
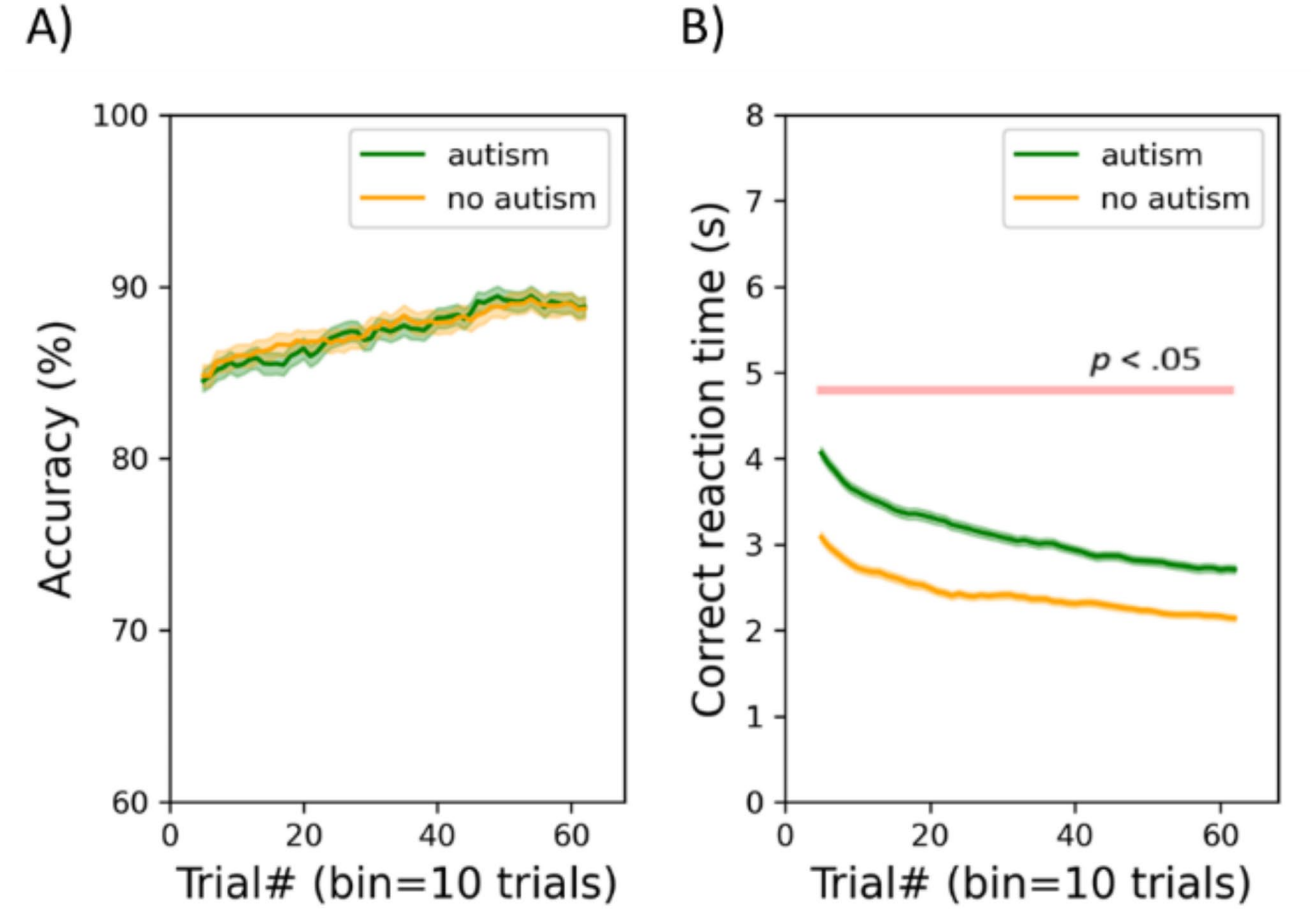
**A**) Mean accuracy per group over the course of the experiment (divided into 10-trial bins). **B**) Mean correct reaction time per group over the course of the experiment. Error bars represent the standard error of the mean. Per bin, significant group differences (*p* <.05, false discovery rate corrected) are indicated by the pink bar

Figure 6a-b illustrates the mean accuracy and mean correct RT over the course of the experiment (i.e., trial number). We divided the trials into bins of 10 and calculated the mean accuracy and mean correct RT following a walking average (see also Van der Burg et al., 2015 for a similar approach). In other words, the first bin corresponds to the mean accuracy and mean correct RT over trial 1–10, the second bin over trial 2–11, etc. The bin size was chosen to allow calculation of a RT or accuracy value for each participant after accounting for lost trials. We conducted repeated-measures ANOVAs on the mean accuracy and mean correct RT with trial bin as a repeated-measures factor and group as a between-subjects variable. Significant group differences per bin are indicated by the pink bar in Fig. 6b.

*Time-course analysis: accuracy.* As in the main analysis, group did not have a significant main effect (*F*(1, 767) = 0.06, *p =*.809, η_p_^2^ = 0.001). Bin had a significant main effect on accuracy (*F*(57, 43719) = 13.293, *p* <.001, η_p_^2^ = 0.017) but did not interact with group (*F*(57, 43719) = 0.391, *p =*.99, η_p_^2^ = 0.001).

*Time-course analysis: reaction time*. As in the main analysis, there was a significant main effect of group (*F*(1, 767) = 139, *p* <.001, η_p_^2^ = 0.153). Bin also had a significant main effect (*F*(57, 43719) = 194.2, *p* <.001 η_p_^2^ = 0.202) and interacted with group (*F*(59, 43719) = 10.3, *p* <.001, η_p_^2^ = 0.013). We conducted two-tailed independent *t*-tests on the mean correct RT in each bin to evaluate the interaction of group and bin, which revealed that there was a significant difference between groups at each bin, which decreased in magnitude over the course of the experiment (seFig. . 6b).

## Experiment 2: Complex Facial Emotion Recognition (RMET)

**Fig. 7:**
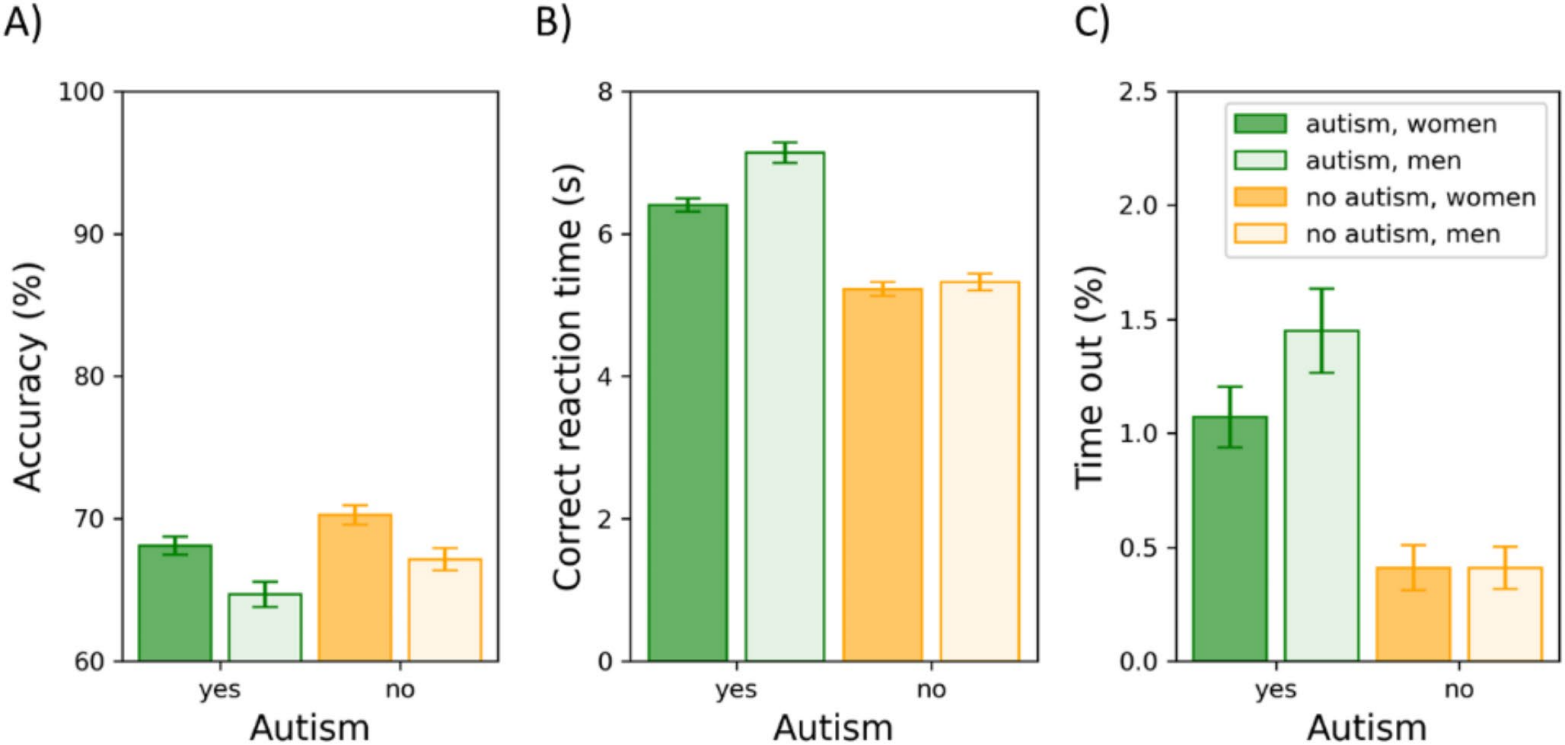
**A**) Mean accuracy as a function of group, according to gender. **B**) Mean correct reaction time as a function of group, according to gender. **C**) Mean percentage time-out as a function of group, according to gender. The error bars reflect the standard error of the mean

Figure 7a shows the mean accuracy (panel A), mean correct RT (panel B) and mean percentage of time-outs (Panel C) by group for women and men. For each dependent variable, we conducted an ANCOVA with group and gender as between-subjects variables and age and ICAR scores as covariates.

*Accuracy.* The ANOVA yielded no effect of group (*F*(1, 903) = 2.442, *p =*.118, η_p_^2^ = 0.003) or interaction between gender and group (*F*(1, 903) = 0.230, *p =*.632, η_p_^2^ = 2.546 × 10^− 4^). However, there was a main effect of gender (*F*(1, 903) = 15.291, *p* <.001, η_p_^2^ = 0.017), such that women (68.9%) performed better than men (65.9%). Age had a negative relationship with accuracy (*F*(1, 903) = 7.660, *p* =.006, η_p_^2^ = 0.008), while ICAR score had a positive one (*F*(1, 903) = 28.425, *p* <.001, η_p_^2^ = 0.031).

*Reaction time.* There was a significant main effect of group (*F*(1, 903) = 71.462, *p <*.001, η_p_^2^ = 0.073), such that autistic participants (6664 ms) performed more slowly than non-autistic ones (5270 ms). There was also a significant main effect of gender (*F*(1, 903) = 10.704, *p* =.001, η_p_^2^ = 0.012), such that women (5955 ms) performed more quickly than men (6214 ms). Age (*F*(1, 903) = 90.585, *p* <.001, η_p_^2^ = 0.091) and ICAR scores (*F*(1, 903) = 30.090, *p* <.001, η_p_^2^ = 0.032) both had positive relationships with RT. There was no significant interaction between gender and group (*F*(1, 903) = 1.208, *p =*.272, η_p_^2^ = 0.001).

*Percentage time-out.* The main effect of group was significant (*F*(1, 903) = 18.774, *p* <.001, η_p_^2^ = 0.020), with autistic participants (1.2%) timing out more frequently than non-autistic ones (0.4%). ICAR (*F*(1, 903) = 7.733, *p* =.006, η_p_^2^ = 0.008) and age (*F*(1, 903) = 7.471, *p* =.006, η_p_^2^ = 0.008) both had positive relationships with the number of time-outs. Neither the main effect of gender (*F*(1, 903) = 1.529, *p* =.217, η_p_^2^ = 0.002) nor the interaction of gender and group (*F*(1, 903) = 0.724, *p* =.395, η_p_^2^ = 8.009 × 10^− 4^) were significant.

**Fig. 8:**
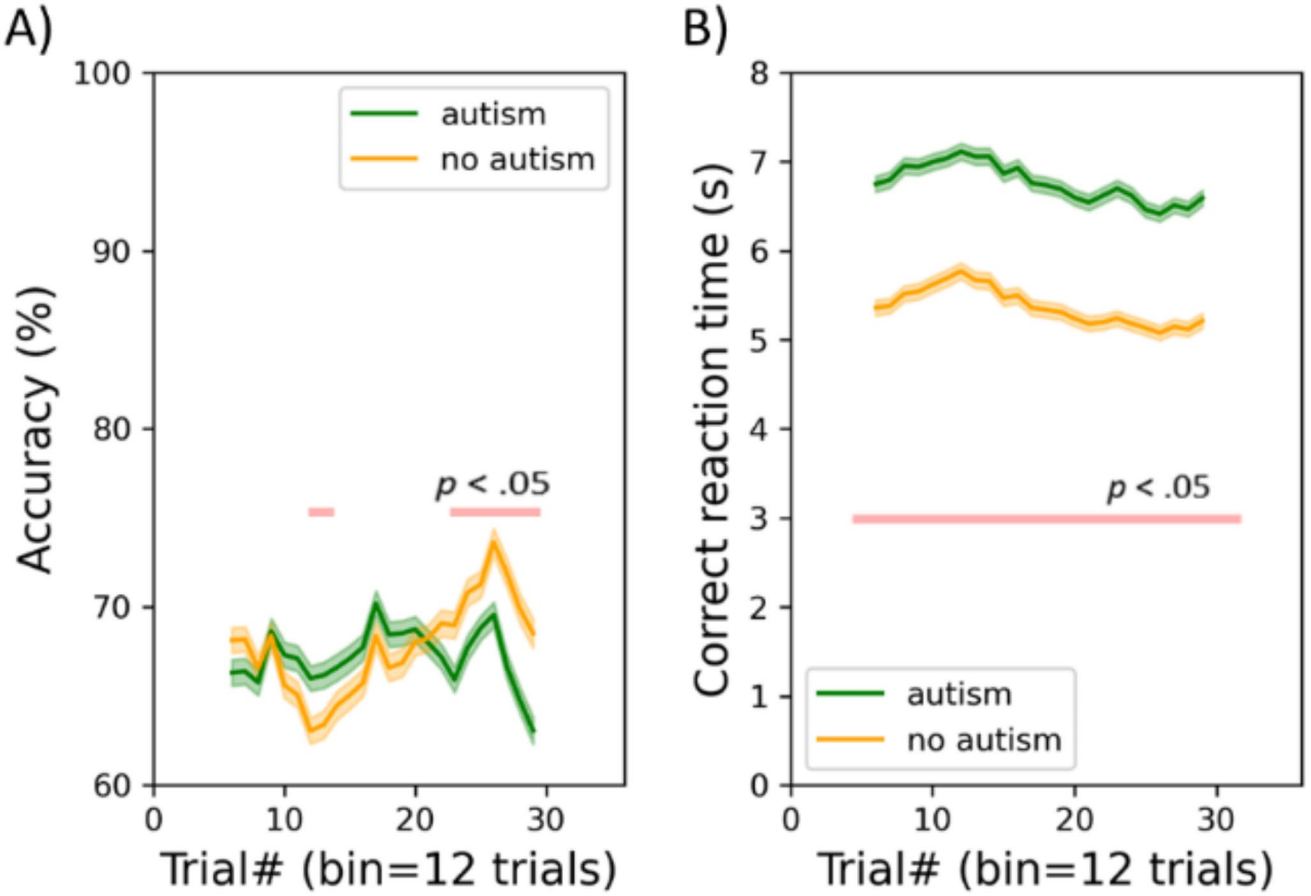
**A**) Mean accuracy per group over the course of the experiment (divided into 12-trial bins). **B**) Mean correct reaction time per group over the course of the experiment. Error-bars represent the standard error of the mean. Per bin, significant group differences (*p* <.05, false discovery rate corrected) are indicated by the pink bars

Figure 8a-b illustrates the mean accuracy and mean correct RT over the course of the experiment (i.e., trial number). We divided the trials into bins of 12 and calculated the mean accuracy and mean correct RT following a walking average, as in the prior experiment. We conducted repeated-measures ANOVAs on the mean accuracy and mean correct RT with trial bin as a repeated-measures factor and group as a between-subjects variable. Significant group differences per bin are indicated by the pink bars in Fig. 8a.

*Time-course analysis: accuracy.* As in the main analysis, there was no significant main effect of group (*F*(1, 907) = 0.481, *p* =.488, η_p_^2^ = 0.001). However, there was a significant effect of bin (*F*(23, 20861) = 23.2, *p* <.001, η_p_^2^ = 0.025) and interaction of bin and group (*F*(23, 20861) = 15.4, *p* <.001, η_p_^2^ = 0.017) on mean accuracy. To investigate the interaction of bin and group, we conducted two-tailed independent *t*-tests on the mean accuracy in each bin, which revealed significant differeces in the bins indicated in Fig. 8a. Autistic participants performed better for a small subset of trials toward the middle of the experiment and worse towards the end.

*Time-course analysis: reaction time.* As in the main analysis, there was a main effect of group (*F*(1, 907) = 139, *p* <.001, η_p_^2^ = 0.133). Bin also had a significant main effect (*F*(23, 20861) = 77.290, *p* <.001, η_p_^2^ = 0.079) but did not interact with group (*F*(23, 20861) = 0.544, *p* =.962, η_p_^2^ = 0.001).

## Experiment 3: Affective Prosody Recognition

**Fig. 9:**
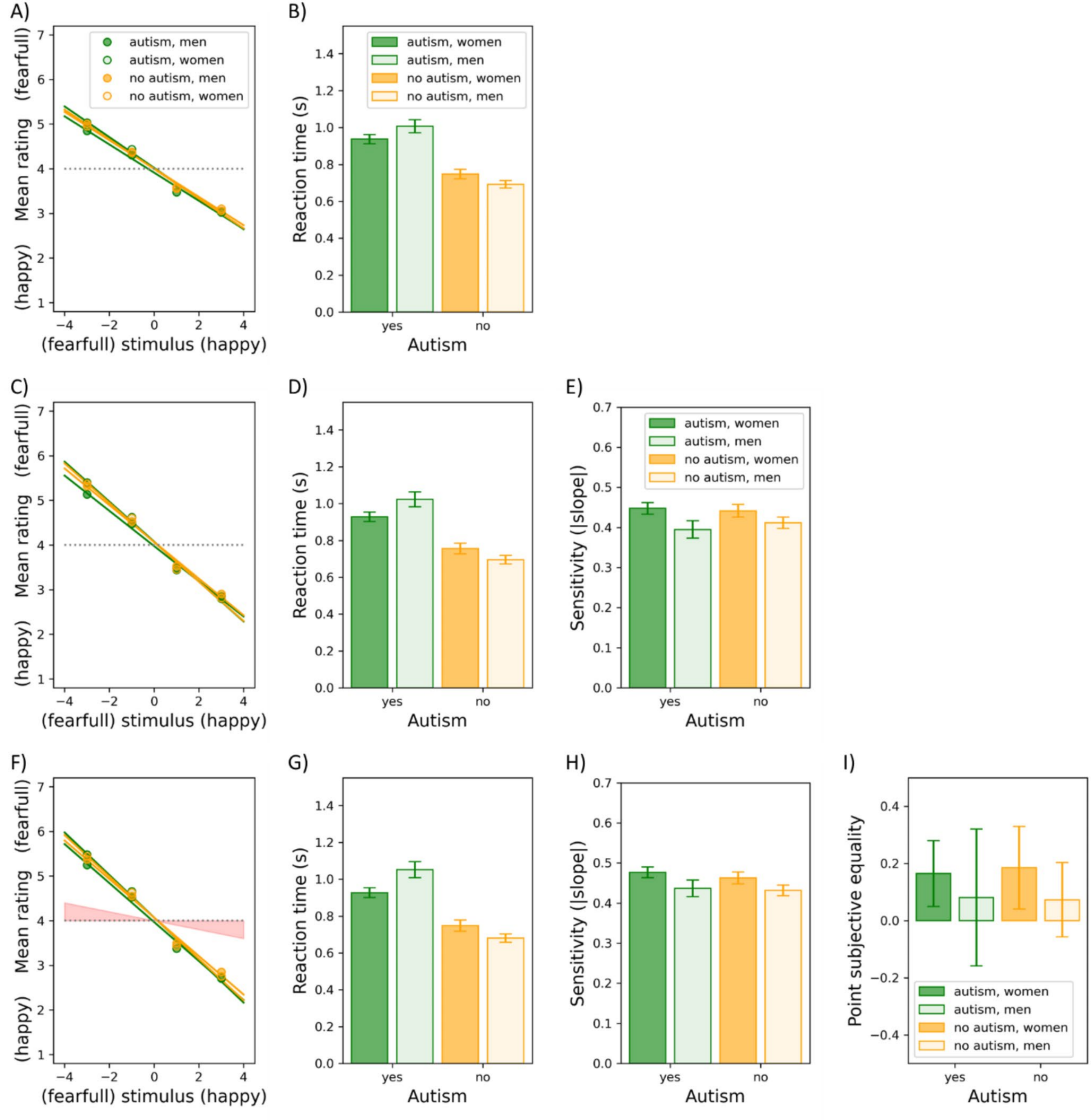
**A**, **C** and **F**) Mean rating as a function of stimulus, group and gender. **B**, **D** and **G**) Mean reaction time as a function of stimulus, group and gender. **E** and **H**) Mean sensitivity as a function of stimulus, group and gender. **I**) Mean point of subjective equality as a function of stimulus, group and gender. Upper row: all participants were included. Middle row: participants with a positive slope were excluded. Lower row: participants with a slope approaching zero were excluded. Error bars reflect the standard error of the mean

For each participant, we fitted a linear function, $\:f\left(x\right)=ax+b$, to the mean rating according to stimulus type to estimate *sensitivity* (i.e., the slope, *a*, of the linear function) to affective prosody. Figure 9a and c, and 9f illustrate a series of representations of this function for different subgroups of autistic and non-autistic participants described below, as well as their point of subjective equality (*PSE*). The PSE is the point on the x-axis where the linear function crosses the dotted line in these panels, corresponding to the stimulus for which participants were most likely to give the middle rating between happy and fearful (4). A bias towards negative or positive interpretations of stimuli would be reflected in the middle rating being given for happier or more fearful stimuli (respectively), rather than near the center of the stimulus continuum (corresponding to 0 on the x axis). Figure 9 also provides comparisons of the mean RT (9b, 9d, and 9 g), sensitivity (9e and 9 h), and PSE (9i) for each group, all of which were analyzed independently.

*Reaction time.* We conducted an ANCOVA on the mean RT with group and gender as between-subject variables and age and ICAR scores as covariates. The ANCOVA yielded a significant main effect of group (*F*(1, 782) = 46.407, *p* <.001, η_p_^2^ = 0.056), such that autistic participants (961 ms) were slower on average than non-autistic ones (719 ms). Additionally, age was positively related to RT (*F*(1, 782) = 9.914, *p* =.002, η_p_^2^ = 0.013). All other *F* ≤ 0.325; all other *p* ≥.119.

As seen in Fig. 9a, a negative slope indicates responses consistent with the typically perceived emotional valence of the stimuli. However, 14.7% of the participants with autism (*n* = 58) and 14.1% of the participants without autism (*n* = 55) showed a positive slope (> 0.0001), indicating that these participants either reversed the keys or misinterpreted the emotions in a systematic manner. These participants were excluded from subsequent analyses. Figure 9c illustrates the mean rating as a function of the stimulus for each group and gender for individuals with a negative slope only. Figure 9d and e illustrate the mean RT and mean sensitivity for those individuals. We conducted an ANCOVA on the mean correct RT and sensitivity with group and gender as between-subject variables and age and ICAR scores as covariates. For RT, the main effect of group was again significant (*F*(1, 782) = 42.897, *p* <.001, η_p_^2^ = 0.060). All other *F* ≤ 4.737; all other *p* ≥.030. Sensitivity did not differ according to any variables (group effect: *F*(1,669) = 0.150, *p* =.689, η_p_^2^ = 2.248 × 10^− 4^; all other *F* ≤ 5.120; all other *p* ≥.024).

We did not conduct a similar analysis on the mean PSE for this group because some participants had a sensitivity close to zero, indicating that they were not able to perform the task well. For many of these participants, fitting functions resulted in an unrealistically large PSE. In order to estimate the PSE for participants who demonstrated sensitivity to the stimuli, we excluded 85 participants with autism (21.4%) and 72 participants without autism (18.5%) with a slope > -0.1 (see the red shading in Fig. 9f). Figure 9 (lower row) illustrates the mean RT, sensitivity, and PSE for the remaining participants. For this group, we conducted ANCOVAs on mean RT, sensitivity, and PSE with group and gender as between-subject variables and age and ICAR scores as covariates. The sensitivity analysis remained consistent, with no significant effects (group effect: *F*(1,625) = 0.980, *p* =.323, η_p_^2^ = 0.002; all other *F* ≤ 4.788; all other *p* ≥.029). The main effect of group on RT remained significant (*F*(1,625) = 52.404, *p* <.001, η_p_^2^ = 0.077). Additionally, group interacted with gender (*F*(1,625) = 7.237, *p* =.007, η_p_^2^ = 0.011) to influence RT. To investigate this interaction, we conducted follow-up two-tailed *t*-tests. While autistic men (1051 ms) were significantly slower (*t*(316) = 2.556, *p* =.011) than autistic women (927 ms), there was no difference between non-autistic men and women (*t*(316) = 1.821, *p* =.070). No factors significantly influenced the PSE (group effect: *F*(1, 625) = 0.003, *p* =.953, η_p_^2^ = 5.558 × 10^− 6^; all other *F* ≤ 1.934; all other *p* ≥.165), and neither group showed evidence of a systematic bias in their interpretations of stimuli.

**Fig. 10:**
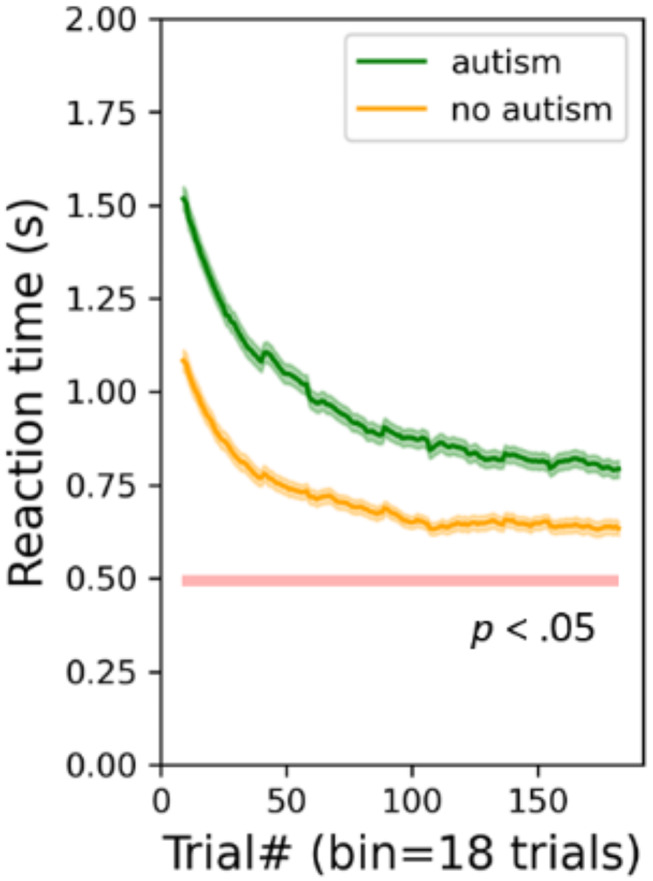
Mean reaction time per group over the course of the experiment (divided into 18-trial bins). Error-bars represent the standard error of the mean. Per bin, significant group differences (*p* <.05, false discovery rate corrected) are indicated by the pink bars

Figure 10 illustrates the mean RT over the course of the experiment (i.e., trial number). We divided the trials into bins of 18 and calculated the mean RT following a walking average, as in the prior experiments. Due to the large number of trials needed to calculate sensitivity and PSE, time series analyses were not possible for these variables. We conducted a repeated-measures ANOVA on the mean RT with trial bin as a repeated-measures factor and group as a between-subjects variable.

*Time-course analysis (reaction time).* As in the main analysis, there was a main effect of group *(F*(1, 786) = 81.4, *p* <.001, η_p_^2^ = 0.094). Bin also had a significant main effect (*F*(173, 135978) = 237.8, *p* <.001, η_p_^2^ = 0.232) and interacted with group (*F*(173, 135978) = 17.6, *p* <.001, η_p_^2^ = 0.022). We conducted two-tailed independent *t*-tests on the mean RT to investigate this interaction and found that they differed significantly at each bin, but the magnitude of the difference decreased over the course of the experiment. Significant group differences per bin are indicated by the pink bar in Fig. 10.

**Note**: Overarching correlational and regression analyses may be found in the Supplementary Materials (Supplementary Fig. 4 and Table 7). Correlational analyses revealed a stronger (*z* = 2.05, *p* =.04) relationship between facial emotion recognition tasks in the autistic group (*r*(292) = 0.383, *p* <.001) than in the non-autistic group (*r*(274) = 0.227, *p* <.001). A logistic regression with all task metrics as predictors showed moderate predictive accuracy (72.1%) for diagnostic category, with RT (but not accuracy/sensitivity) on each task contributing significantly to the model.

## Discussion

Across tasks, our results follow a consistent pattern of intact but protracted emotion recognition among autistic participants. Demographic (i.e., age, gender, and IQ) and task (i.e., stimulus type/complexity) features had generally similar influences on performance across groups. Correlational analyses revealed a closer relationship between facial emotion recognition measures in the autism group. Finally, a logistic regression combining results from all three tasks confirmed they could distinguish between groups with a reasonable degree of reliability, but that only RT differences were of use in doing so. Together, these findings offer a clearer picture of multimodal emotion recognition abilities in autism and the best ways of capturing the differences that exist between autistic and non-autistic individuals.

Our primary finding was that RT but not accuracy/sensitivity (as confirmed in our Bayesian follow-up analyses; see Supplementary Tables 1–6) differed consistently between groups across measures. This resonates with previous findings that autistic individuals can perform as well as non-autistic ones in recognizing both facial and prosodic emotions when given sufficient time (Castelli, 2005; Leung et al., 2023; Matsumoto et al., 2016). On the other hand, it is at odds with the meta-analyses that have reported significant evidence of a difference between groups in accuracy, at least with facial emotion recognition (Lozier et al., 2014; Uljarevic & Hamilton, 2013; Yeung, 2022). This discrepancy may be partially explained by the fact that the meta-analyses included some studies that imposed stricter time constraints on stimulus presentation or response times, given the long RTs we detected among autistic participants. However, there are also studies (Baron-Cohen et al., 2001; Meyer-Lindenberg et al., 2022; Peñuelas-Calvo et al., 2019; Sucksmith et al., 2013) using the same facial stimuli as ours without these constraints that have detected a group effect on accuracy. This means that the lack of strict time constraints alone is not a satisfactory explanation for why we did not find differences in accuracy. The comparability of designs in these cases suggests that sample characteristics may contribute to the differences in outcomes, particularly given that Meyer-Lindenberg et al. (2022) found that the disparity between groups was driven by a low-performing subgroup of autistic participants.

Our results speak directly to the influence of key demographic factors that might be expected to distinguish these samples. We found that women from both groups tended to process facial emotions more quickly and effectively than men, consistent with prior literature (Kapitanović et al., 2022; Wingenbach et al., 2018). Furthermore, we were the first to replicate the findings of diminished emotion recognition abilities among older adults seen in the general population (Ruffman et al., 2008) in an autistic sample. Older participants from both groups were less accurate on the facial emotion recognition tasks and slower on all tasks. Higher estimated IQ also correlated with greater accuracy in both facial emotion recognition tasks but slower responses for complex emotion recognition (RMET). Crucially, these factors did not interact with group (with the singular exception of gender differences in RT on the prosody task only being present for autistic participants). This reflects a similar profile of demographic influences on emotion processing among autistic and non-autistic individuals, and it suggests that these specific factors are not responsible for the difference in outcomes between our study and some previous ones.

Task demands also seemed to have a very similar influence upon the groups. Autistic and non-autistic participants struggled with recognizing certain basic emotional categories (particularly fearful faces) to a similar degree in terms of accuracy (although group differences in RT were larger for certain categories). The more complex emotional stimuli in RMET were harder to recognize for both groups, but they still did not differ significantly in their accuracy. Performance over the course of the experiments also followed a generally similar trajectory, with accuracy steadily improving on KDEF and RT declining over time for both groups in all tasks. It is worth noting that the differences between groups in RT decreased in magnitude over the course of KDEF and the prosody task. This may be because the general decline in RT left less room for a group effect. Alternatively, given findings that autistic individuals show diminished cognitive flexibility (Lage et al., 2024), larger differences on earlier trials may have been due to a greater cost of task switching for the autistic group. Additionally, on RMET, despite comparable accuracy overall, autistic participants performed better for a small subset of trials towards the middle of the experiment and worse towards the end. However, given the findings that autistic and non-autistic individuals find different trials more difficult (Lombardo et al., 2016), this was likely due (at least in part) to the fact that the order of stimuli in the RMET is not randomized. As such, these findings cannot be taken as evidence of different rates of attrition on RMET, and future research should further investigate what about certain trials makes them more sensitive to differences between autistic and non-autistic individuals.

These broad similarities in the influences of demographic and task features on performance between groups suggest that the cause of the heterogeneity in findings may lie elsewhere. One factor that could play a role here is the inevitable variability introduced by small sample sizes, which are still common in the literature. It is notable that our sample is far larger than is standard in the field; however, others using the same or similar tasks have produced different results with fairly large groups (Meyer-Lindenberg et al., 2022; Sucksmith et al., 2013; Wilson et al., 2014). One frequently discussed explanation for these discrepancies is that shifting diagnostic criteria and an increase in adult diagnoses may have contributed to diminishing effect sizes in the autism literature (Rødgaard et al., 2019). It is suspected that those diagnosed later in life may have less noticeable behavioral differences, a notion that is supported by findings that more pronounced autistic traits predict a younger age of diagnosis (Hrdlicka et al., 2023). Given that the NAR cohort is disproportionately composed of adults diagnosed later in life (Scheeren et al., 2022), and other studies do not report age of diagnosis, it is possible that this unaddressed demographic factor may play a role. However, age of diagnosis and chronological age are nearly inextricable factors given how closely they overlap (*r* =.91 in Scheeren et al., 2022). The fact that chronological age also played an important role in all of our measures means that any investigation of the influence of age of diagnosis would be confounded by it without proper control. As such, future research should focus on evaluating this possibility with targeted recruitment of participants to allow an age-matched comparison of those diagnosed early and late in life.

In addition to our findings within measures, we detected evidence of a correlation in accuracy between the two facial emotion recognition tasks for both groups, which was significantly stronger for the autistic participants (see Supplementary Fig. 4). Due to the fact that the groups did not differ significantly in accuracy on either facial emotion recognition measure, the tighter correlation between them among autistic participants cannot be explained by common impairment. However, the longer mean RTs on these tasks (as well as the more frequent time-outs on RMET) suggest that they may have been more demanding for autistic participants and/or that they may have used more time-consuming strategies to reach similar levels of performance (see: Harms et al., 2010; Leung et al., 2023; Matsumoto et al., 2016; Yeung, 2022). A greater reliance on specific rules for categorization involving attention to featural details (Yeung, 2022) or verbal mediation (Harms et al., 2010) may explain both the protracted RTs and the stronger correlation between measures in the autism group if the strategies employed are similar across them. Alternatively, RT differences could arise (at least in part) from factors not directly related to emotion recognition, such as slower motor response speed (Fournier et al., 2010; Morrison et al., 2018) or meta-cognitive differences (van der Plas et al., 2023). As such, despite the stronger correlation between facial emotion recognition RTs in the autism group, it would be premature to conclude that they exclusively reflect slower processing of emotions themselves.

Together, our dependent variables demonstrated a reasonable degree of accuracy (72.1%) in predicting autism in our logistic regression (depicted in Supplementary Table 7). More sophisticated approaches using machine learning and a larger battery of online behavioral tasks or neurophysiological data have achieved a predictive accuracy of 78% (Dubey et al., 2024) and ranging from 48.3 to 97% (Liu et al., 2021), respectively. So, although this approach does not match the best alternatives, it does provide evidence that performance in simple online emotion-related tasks can contribute to computing the likelihood of an individual being autistic. However, because age correlated positively with RT, and women tended to have lower RTs on the facial tasks, the model’s predictive accuracy may be influenced by disparities in these factors between groups (given that the autistic group was older and included proportionally more women). Individual matching on these variables meant losing the majority of our data, but future research should target recruitment to provide a more reliable estimate of how well emotion recognition measures can predict diagnosis given the proof of concept these results provide.

Although aspects of all three tasks had significant predictive accuracy, it appears as though RMET and KDEF RT most reliably distinguished between groups, consistent with the fact that they produced the largest estimated group effect sizes. The fact that only RT measures were significant contributors to the model suggests that they are much more sensitive to differences between autistic and non-autistic individuals than accuracy. This is especially true when looking at more fine-grained distinctions, like the effect of emotional category in KDEF, where an interaction with group only emerged in the RT data. This augments the evidence offered by the logistic regression, indicating that for both the purposes of distinguishing groups and detecting subtle differences in emotion processing, RT may be the most informative metric.

Our study is subject to limitations, particularly with regard to stimulus sets and generalizability. We focused on the most popular measures of facial emotion recognition, but the inclusion of wider stimulus sets including varying stimulus intensities may have led to greater sensitivity to differences between groups (Law Smith et al., 2010; Song & Hakoda, 2018). Furthermore, the degree to which our findings of comparable accuracy in facial emotion recognition generalize to interpersonal interactions remains to be seen. Some studies have shown greater differences using video stimuli (Enticott et al., 2014; Evers et al., 2015), and questions about the extents to which emotion recognition in images correlates with that in videos and in real life warrant further research. Variation of stimulus complexity and dynamics could allow insight into whether the RT differences observed here could translate into accuracy differences in more fast-paced, naturalistic contexts.

There are also specific features of our sample to keep in mind when interpreting these findings. While our control participants reported that they had not received an autism diagnosis, it is possible that some undiagnosed individuals participated, as in any study investigating autism. However, rather than restricting our control group (for example, by imposing AQ cut-offs), we chose to simply compare those with and without diagnoses. Imposing cut-offs based on autistic traits would have meant confining analyses to subgroups within either population that might be easier to distinguish in our logistic regression and where differences may be more likely to be detected than in the general populations. Our approach was more conservative, and the comparison of individuals with/without diagnoses should have greater ecological validity, given the fact that the distributions of autistic traits in community samples from these groups overlap considerably. Despite this conservative approach, the vast disparity between groups in mean AQ scores suggests that autistic traits differed significantly enough between them to detect associated differences in emotion recognition. There is also the possibility that the groups showed some differences in motivation, given that NAR participants are volunteers and Prolific participants are paid; however, we provided compensation to both groups to minimize this possibility. Finally, the NAR cohort is disproportionately comprised of well-educated individuals in western Europe with normal to high IQs, which is reflected in the fact that they did not differ significantly from our control group in ICAR-16 scores (and our control group performed similarly to other large online cohorts, such as Merz et al., 2022). In the future, our findings need to be replicated across a range of intellectual abilities and diverse global regions to assess generalizability. However, a benefit of the sampling bias in the current study is that we can focus on differences in emotion processing that are not confounded by difficulties understanding the tasks or words presented. Additionally, we had parity in mean IQ between groups, and it was not found to interact with group on any measures, suggesting that it was not a determining factor in our findings.

In conclusion, our multimodal exploration of emotion recognition has revealed much about the ways in which autistic and non-autistic individuals differ and the comparable influence of task and demographic features on performance between groups. We found compelling evidence that RTs are much more informative measures than accuracy/sensitivity in both facial and prosodic emotion recognition, better distinguishing groups and offering greater insight into subtle differences between them. These findings have theoretical implications regarding the nature of emotion recognition in autism, where adults appear to function differently but as effectively when provided sufficient time. They also have clear practical implications, demonstrating that emotion recognition measures may be useful indicators of the likelihood an individual is autistic (but possibly only when RT data is collected). Moreover, they suggest that emotion recognition training might be better tailored to the needs of autistic individuals if it focuses on efficiency over accuracy (given evidence that both can show improvement, see Russo-Ponsaran et al., 2016), especially considering the fast-paced demands of daily social interaction. In sum, these findings are encouraging in what they reflect of the ability of autistic individuals to perform in an area often thought to be one of considerable impairment, and they are promising in the directions they offer for improving our tools to identify, understand, and help those with autism.

## Electronic supplementary material

Below is the link to the electronic supplementary material.

Supplementary Material 1

## Footnotes

1 We alternate between person-first and identity-first language to address the varying preferences of the international autism community, as recommended in Buijsman et al. (2023).

2 In a follow-up paper, we aim to measure potential inter-trial effects on prosodic emotion recognition (as in Van der Burg et al., 2024) for individuals with and without autism. No-go trials were for these analyses. However, measuring serial dependence is not within the scope of the present article, so we have simply reported findings for go trials across previous trial conditions.

3 Note: the degrees of freedom differ here because some participants had an accuracy of 0 for fearful (18 autistic and 27 non-autistic participants) or sad (1 non-autistic participant) stimuli, resulting in their exclusion from the mean correct RT by category analysis.
