# Supplementary material for: Intact but Protracted Facial and Prosodic Emotion Recognition Among Autistic Adults
Source: J Autism Dev Disord. 2025 Mar 27;56(9):3403–20. doi: 10.1007/s10803-025-06786-z (PMC13427989; doi:10.1007/s10803-025-06786-z)
Supplement: Supplementary file 1 — Supplementary Material 1 [file 10803_2025_6786_MOESM1_ESM.docx]

**Supplementary Materials**

**Intact but Protracted Facial and Prosodic Emotion Recognition in Autistic Adults**


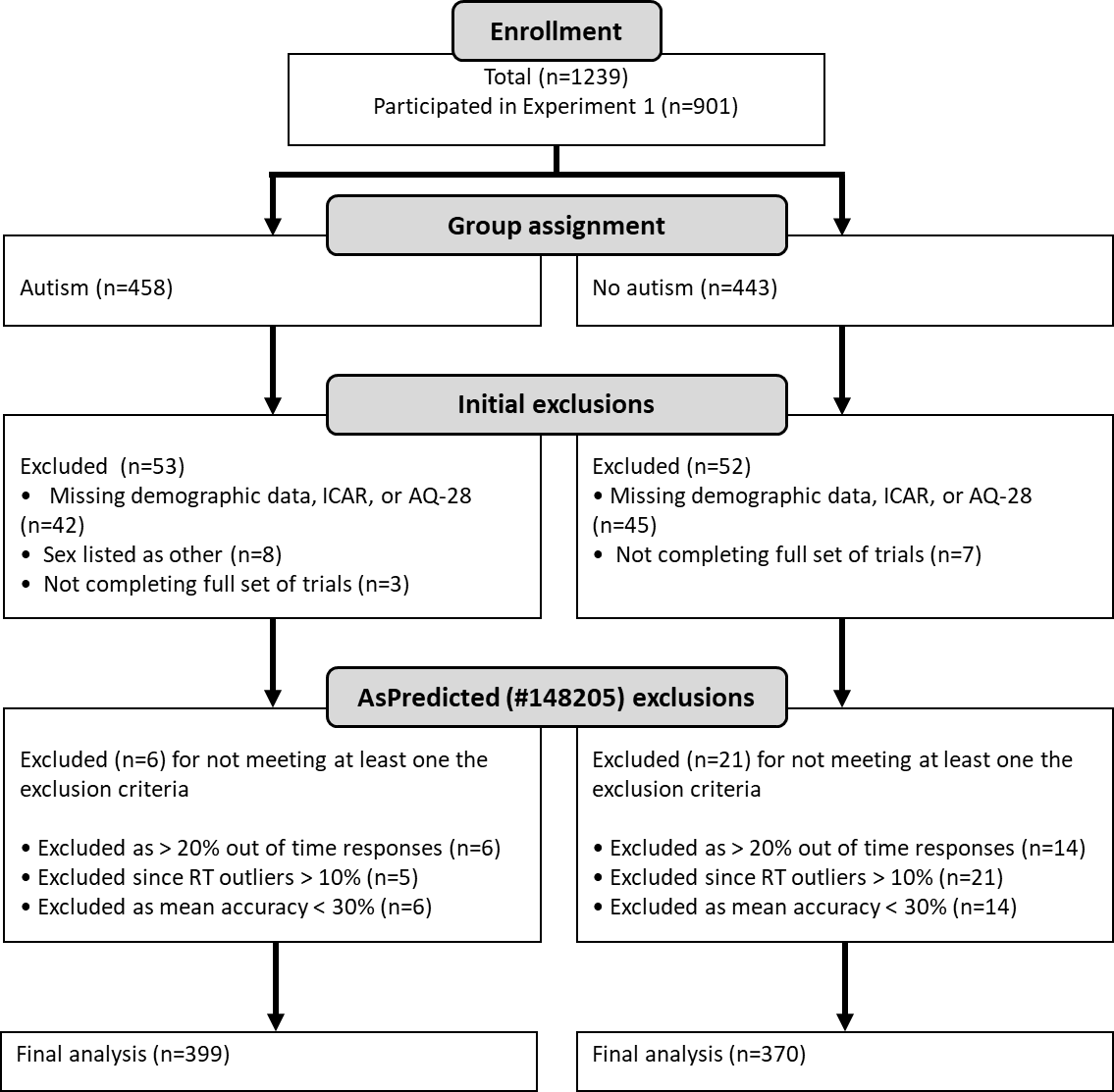


Supplementary Figure 1: Participant flow chart for Experiment 1.


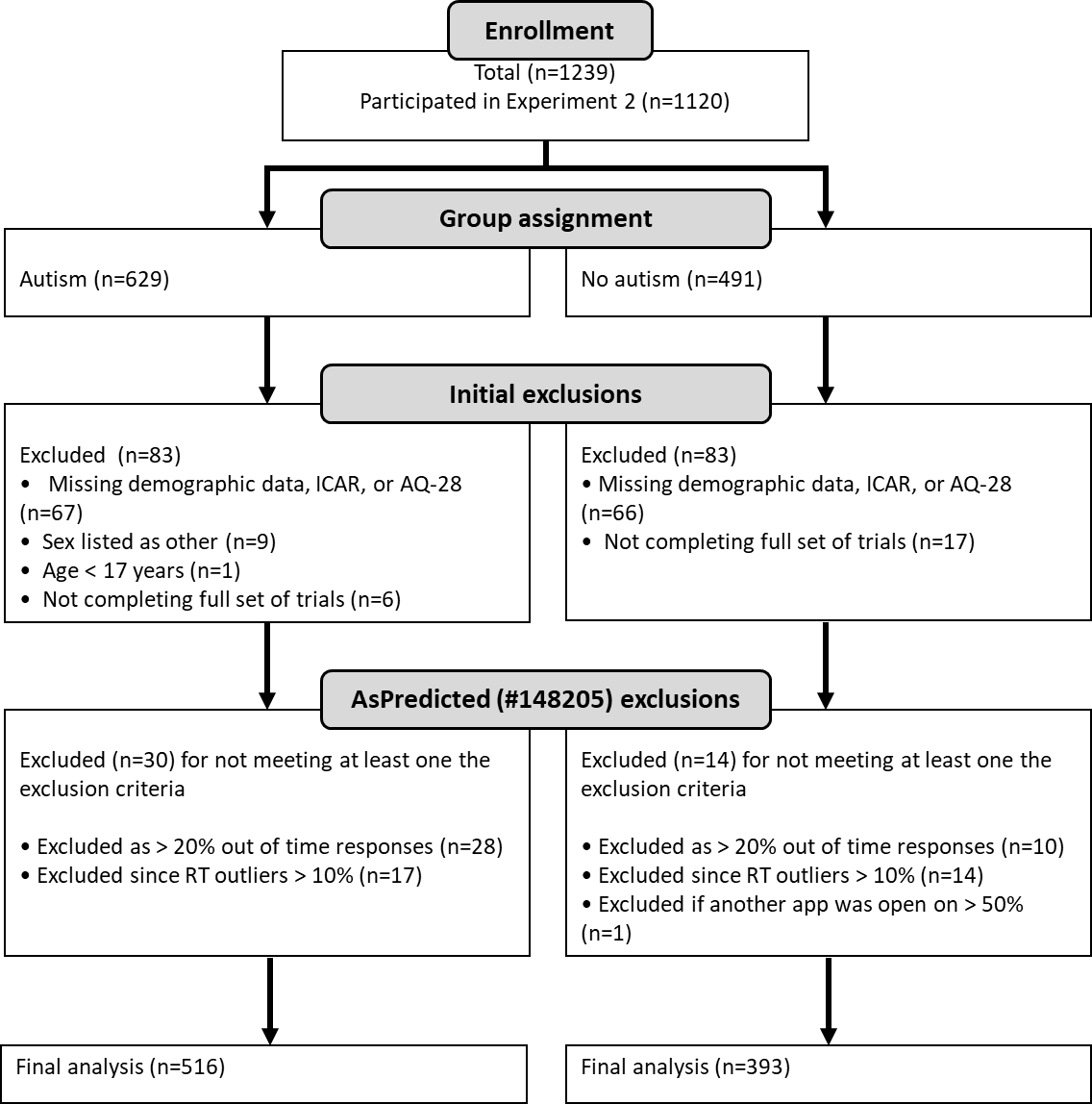


Supplementary Figure 2: Participant flow chart for Experiment 2.


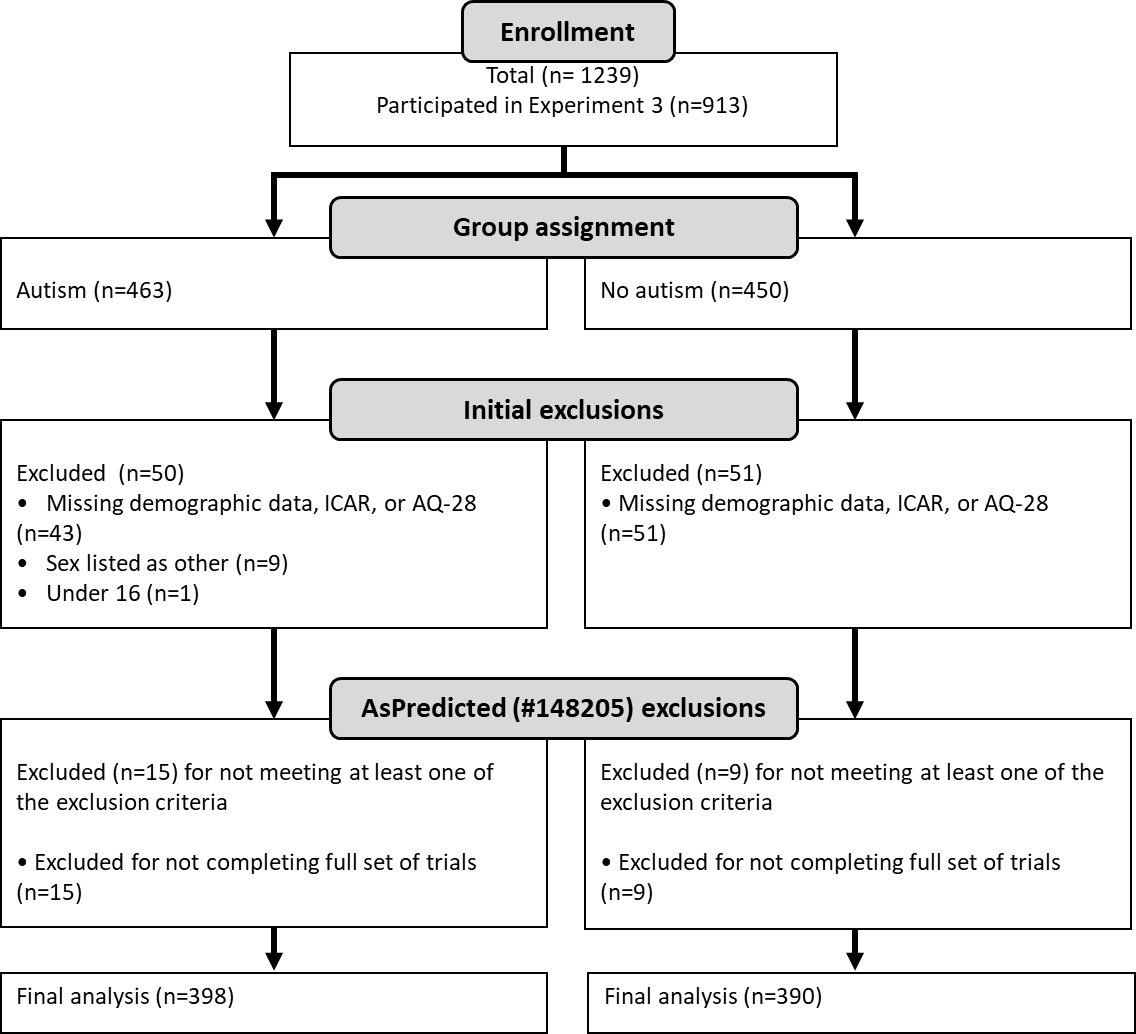


Supplementary Figure 3: Participant flow chart for Experiment 3.

Supplementary Table 1: Results of Bayesian analysis of covariance on mean accuracy for the Karolinska Directed Emotional Faces task with group and gender as between-subjects variables and age and International Cognitive Ability Resource intelligence quotient scores as covariates. This analysis was conducted using uniform model priors and default priors on coefficients (*r* scale prior width = 0.5 for fixed effects and 0.354 for covariates).

| **Model Comparison** | | | | | | | | | | | |
| --- | --- | --- | --- | --- | --- | --- | --- | --- | --- | --- | --- |
| **Models** | | **P(M)** | | **P(M\|data)** | | **BF_M_** | | **BF_10_** | | **error %** | |
| Null model |  | 0.050 |  | 1.431×10^-4^ |  | 0.003 |  | 1.000 |  |  |  |
| Age + ICAR + Gender |  | 0.050 |  | 0.738 |  | 53.573 |  | 5160.354 |  | 4.400 |  |
| ICAR + Gender |  | 0.050 |  | 0.067 |  | 1.366 |  | 468.872 |  | 1.009 |  |
| ('Group', '') + Age + ICAR + Gender |  | 0.050 |  | 0.066 |  | 1.333 |  | 458.249 |  | 3.340 |  |
| Age + ICAR |  | 0.050 |  | 0.056 |  | 1.127 |  | 391.416 |  | 0.012 |  |
| Age + Gender |  | 0.050 |  | 0.030 |  | 0.592 |  | 211.364 |  | 1.751 |  |
| ('Group', '') + Age + ICAR + Gender + ('Group', '') ✻  Gender |  | 0.050 |  | 0.010 |  | 0.198 |  | 71.934 |  | 4.452 |  |
| ('Group', '') + ICAR + Gender |  | 0.050 |  | 0.009 |  | 0.176 |  | 64.175 |  | 2.886 |  |
| ('Group', '') + Age + ICAR |  | 0.050 |  | 0.007 |  | 0.125 |  | 45.692 |  | 4.247 |  |
| ICAR |  | 0.050 |  | 0.005 |  | 0.090 |  | 32.992 |  | 0.005 |  |
| Gender |  | 0.050 |  | 0.004 |  | 0.082 |  | 30.111 |  | 7.591×10^-4^ |  |
| ('Group', '') + Age + Gender |  | 0.050 |  | 0.003 |  | 0.054 |  | 19.960 |  | 2.257 |  |
| ('Group', '') + ICAR + Gender + ('Group', '') ✻  Gender |  | 0.050 |  | 0.002 |  | 0.042 |  | 15.498 |  | 4.852 |  |
| Age |  | 0.050 |  | 0.001 |  | 0.020 |  | 7.409 |  | 0.005 |  |
| ('Group', '') + Gender |  | 0.050 |  | 5.406×10^-4^ |  | 0.010 |  | 3.779 |  | 1.739 |  |
| ('Group', '') + ICAR |  | 0.050 |  | 4.039×10^-4^ |  | 0.008 |  | 2.823 |  | 1.648 |  |
| ('Group', '') + Age + Gender + ('Group', '') ✻  Gender |  | 0.050 |  | 3.919×10^-4^ |  | 0.007 |  | 2.739 |  | 2.350 |  |
| ('Group', '') + Age |  | 0.050 |  | 1.529×10^-4^ |  | 0.003 |  | 1.069 |  | 5.703 |  |
| ('Group', '') + Gender + ('Group', '') ✻  Gender |  | 0.050 |  | 1.389×10^-4^ |  | 0.003 |  | 0.971 |  | 11.313 |  |
| ('Group', '') |  | 0.050 |  | 1.227×10^-5^ |  | 2.331×10^-4^ |  | 0.086 |  | 0.224 |  |
|  | | | | | | | | | | | |

Supplementary Table 2: Results of Bayesian analysis of covariance on mean correct reaction time for the Karolinska Directed Emotional Faces task with group and gender as between-subjects variables and age and International Cognitive Ability Resource intelligence quotient scores as covariates. This analysis was conducted using uniform model priors and default priors on coefficients (*r* scale prior width = 0.5 for fixed effects and 0.354 for covariates).

| **Model Comparison** | | | | | | | | | | | |
| --- | --- | --- | --- | --- | --- | --- | --- | --- | --- | --- | --- |
| **Models** | | **P(M)** | | **P(M\|data)** | | **BF_M_** | | **BF_10_** | | **error %** | |
| Null model |  | 0.050 |  | 2.315×10^-63^ |  | 4.399×10^-62^ |  | 1.000 |  |  |  |
| ('Group', '') + Age |  | 0.050 |  | 0.457 |  | 16.002 |  | 1.975×10^+62^ |  | 1.051 |  |
| ('Group', '') + Age + Gender |  | 0.050 |  | 0.354 |  | 10.397 |  | 1.528×10^+62^ |  | 1.419 |  |
| ('Group', '') + Age + Gender + ('Group', '') ✻  Gender |  | 0.050 |  | 0.100 |  | 2.119 |  | 4.333×10^+61^ |  | 6.797 |  |
| ('Group', '') + Age + ICAR |  | 0.050 |  | 0.042 |  | 0.842 |  | 1.834×10^+61^ |  | 1.092 |  |
| ('Group', '') + Age + ICAR + Gender |  | 0.050 |  | 0.036 |  | 0.718 |  | 1.572×10^+61^ |  | 1.184 |  |
| ('Group', '') + Age + ICAR + Gender + ('Group', '') ✻  Gender |  | 0.050 |  | 0.010 |  | 0.192 |  | 4.312×10^+60^ |  | 1.675 |  |
| Age |  | 0.050 |  | 1.048×10^-9^ |  | 1.990×10^-8^ |  | 4.524×10^+53^ |  | 0.005 |  |
| Age + Gender |  | 0.050 |  | 1.114×10^-10^ |  | 2.116×10^-9^ |  | 4.811×10^+52^ |  | 1.621 |  |
| Age + ICAR |  | 0.050 |  | 1.045×10^-10^ |  | 1.986×10^-9^ |  | 4.515×10^+52^ |  | 0.003 |  |
| Age + ICAR + Gender |  | 0.050 |  | 1.183×10^-11^ |  | 2.249×10^-10^ |  | 5.111×10^+51^ |  | 1.139 |  |
| ('Group', '') + Gender + ('Group', '') ✻  Gender |  | 0.050 |  | 2.247×10^-31^ |  | 4.269×10^-30^ |  | 9.705×10^+31^ |  | 4.049 |  |
| ('Group', '') + ICAR + Gender + ('Group', '') ✻  Gender |  | 0.050 |  | 2.306×10^-32^ |  | 4.381×10^-31^ |  | 9.958×10^+30^ |  | 5.480 |  |
| ('Group', '') + Gender |  | 0.050 |  | 6.674×10^-34^ |  | 1.268×10^-32^ |  | 2.883×10^+29^ |  | 1.915 |  |
| ('Group', '') + ICAR + Gender |  | 0.050 |  | 1.184×10^-34^ |  | 2.250×10^-33^ |  | 5.115×10^+28^ |  | 42.526 |  |
| ('Group', '') |  | 0.050 |  | 1.013×10^-34^ |  | 1.925×10^-33^ |  | 4.377×10^+28^ |  | 2.484×10^-35^ |  |
| ('Group', '') + ICAR |  | 0.050 |  | 9.406×10^-36^ |  | 1.787×10^-34^ |  | 4.063×10^+27^ |  | 3.543 |  |
| ICAR |  | 0.050 |  | 2.713×10^-64^ |  | 5.154×10^-63^ |  | 0.117 |  | 0.002 |  |
| Gender |  | 0.050 |  | 2.476×10^-64^ |  | 4.704×10^-63^ |  | 0.107 |  | 0.180 |  |
| ICAR + Gender |  | 0.050 |  | 2.900×10^-65^ |  | 5.511×10^-64^ |  | 0.013 |  | 1.153 |  |
|  | | | | | | | | | | | |

Supplementary Table 3: Results of Bayesian analysis of covariance on mean accuracy for the Reading the Mind in the Eyes Test with group and gender as between-subjects variables and age and International Cognitive Ability Resource intelligence quotient scores as covariates. This analysis was conducted using uniform model priors and default priors on coefficients (*r* scale prior width = 0.5 for fixed effects and 0.354 for covariates).

| **Model Comparison** | | | | | | | | | | | |
| --- | --- | --- | --- | --- | --- | --- | --- | --- | --- | --- | --- |
| **Models** | | **P(M)** | | **P(M\|data)** | | **BF_M_** | | **BF_10_** | | **error %** | |
| Null model |  | 0.050 |  | 2.437×10^-10^ |  | 4.630×10^-9^ |  | 1.000 |  |  |  |
| Age + ICAR + Gender |  | 0.050 |  | 0.656 |  | 36.220 |  | 2.692×10^+9^ |  | 1.320 |  |
| Group + Age + ICAR + Gender |  | 0.050 |  | 0.238 |  | 5.928 |  | 9.759×10^+8^ |  | 1.824 |  |
| Group + ICAR + Gender |  | 0.050 |  | 0.051 |  | 1.018 |  | 2.088×10^+8^ |  | 3.213 |  |
| Group + Age + ICAR + Gender + Group ✻  Gender |  | 0.050 |  | 0.029 |  | 0.569 |  | 1.193×10^+8^ |  | 2.319 |  |
| Age + ICAR |  | 0.050 |  | 0.012 |  | 0.233 |  | 4.966×10^+7^ |  | 0.002 |  |
| ICAR + Gender |  | 0.050 |  | 0.007 |  | 0.125 |  | 2.688×10^+7^ |  | 0.929 |  |
| Group + ICAR + Gender + Group ✻  Gender |  | 0.050 |  | 0.006 |  | 0.107 |  | 2.305×10^+7^ |  | 1.781 |  |
| Group + Age + ICAR |  | 0.050 |  | 0.002 |  | 0.035 |  | 7.612×10^+6^ |  | 1.399 |  |
| Group + ICAR |  | 0.050 |  | 1.398×10^-4^ |  | 0.003 |  | 573559.408 |  | 0.980 |  |
| ICAR |  | 0.050 |  | 7.560×10^-5^ |  | 0.001 |  | 310257.596 |  | 0.006 |  |
| Age + Gender |  | 0.050 |  | 7.598×10^-6^ |  | 1.444×10^-4^ |  | 31181.816 |  | 1.823 |  |
| Group + Age + Gender |  | 0.050 |  | 2.329×10^-6^ |  | 4.426×10^-5^ |  | 9560.347 |  | 1.452 |  |
| Group + Gender |  | 0.050 |  | 3.318×10^-7^ |  | 6.305×10^-6^ |  | 1361.931 |  | 1.010 |  |
| Group + Age + Gender + Group ✻  Gender |  | 0.050 |  | 2.544×10^-7^ |  | 4.834×10^-6^ |  | 1044.151 |  | 2.842 |  |
| Age |  | 0.050 |  | 7.404×10^-8^ |  | 1.407×10^-6^ |  | 303.865 |  | 0.006 |  |
| Group + Gender + Group ✻  Gender |  | 0.050 |  | 6.153×10^-8^ |  | 1.169×10^-6^ |  | 252.507 |  | 40.683 |  |
| Gender |  | 0.050 |  | 4.397×10^-8^ |  | 8.355×10^-7^ |  | 180.465 |  | 1.307×10^-4^ |  |
| Group + Age |  | 0.050 |  | 9.681×10^-9^ |  | 1.839×10^-7^ |  | 39.730 |  | 1.108 |  |
| Group |  | 0.050 |  | 4.051×10^-10^ |  | 7.697×10^-9^ |  | 1.663 |  | 0.013 |  |
|  | | | | | | | | | | | |

Supplementary Table 4: Results of Bayesian analysis of covariance on mean correct reaction time for the Reading the Mind in the Eyes Test with group and gender as between-subjects variables and age and International Cognitive Ability Resource intelligence quotient scores as covariates. This analysis was conducted using uniform model priors and default priors on coefficients (*r* scale prior width = 0.5 for fixed effects and 0.354 for covariates).

| **Model Comparison** | | | | | | | | | | | |
| --- | --- | --- | --- | --- | --- | --- | --- | --- | --- | --- | --- |
| **Models** | | **P(M)** | | **P(M\|data)** | | **BF_M_** | | **BF_10_** | | **error %** | |
| Null model |  | 0.050 |  | 8.400×10^-55^ |  | 1.596×10^-53^ |  | 1.000 |  |  |  |
| Group + Age + ICAR + Gender |  | 0.050 |  | 0.804 |  | 77.958 |  | 9.572×10^+53^ |  | 1.245 |  |
| Group + Age + ICAR + Gender + Group ✻  Gender |  | 0.050 |  | 0.153 |  | 3.440 |  | 1.825×10^+53^ |  | 1.705 |  |
| Group + Age + ICAR |  | 0.050 |  | 0.043 |  | 0.847 |  | 5.080×10^+52^ |  | 0.896 |  |
| Group + Age + Gender |  | 0.050 |  | 4.488×10^-6^ |  | 8.527×10^-5^ |  | 5.343×10^+48^ |  | 1.715 |  |
| Group + Age + Gender + Group ✻  Gender |  | 0.050 |  | 7.923×10^-7^ |  | 1.505×10^-5^ |  | 9.432×10^+47^ |  | 3.470 |  |
| Group + Age |  | 0.050 |  | 5.794×10^-7^ |  | 1.101×10^-5^ |  | 6.897×10^+47^ |  | 0.685 |  |
| Age + ICAR |  | 0.050 |  | 3.101×10^-14^ |  | 5.892×10^-13^ |  | 3.692×10^+40^ |  | 8.483×10^-4^ |  |
| Age + ICAR + Gender |  | 0.050 |  | 1.616×10^-14^ |  | 3.071×10^-13^ |  | 1.924×10^+40^ |  | 1.072 |  |
| Age |  | 0.050 |  | 4.458×10^-19^ |  | 8.471×10^-18^ |  | 5.308×10^+35^ |  | 0.005 |  |
| Group + ICAR + Gender + Group ✻  Gender |  | 0.050 |  | 2.733×10^-19^ |  | 5.194×10^-18^ |  | 3.254×10^+35^ |  | 1.516 |  |
| Age + Gender |  | 0.050 |  | 1.433×10^-19^ |  | 2.722×10^-18^ |  | 1.705×10^+35^ |  | 1.381 |  |
| Group + ICAR + Gender |  | 0.050 |  | 4.075×10^-20^ |  | 7.742×10^-19^ |  | 4.851×10^+34^ |  | 1.174 |  |
| Group + ICAR |  | 0.050 |  | 1.133×10^-22^ |  | 2.153×10^-21^ |  | 1.349×10^+32^ |  | 0.820 |  |
| Group + Gender + Group ✻  Gender |  | 0.050 |  | 5.440×10^-23^ |  | 1.034×10^-21^ |  | 6.476×10^+31^ |  | 2.046 |  |
| Group + Gender |  | 0.050 |  | 1.312×10^-23^ |  | 2.493×10^-22^ |  | 1.562×10^+31^ |  | 3.103 |  |
| Group |  | 0.050 |  | 1.037×10^-25^ |  | 1.970×10^-24^ |  | 1.234×10^+29^ |  | 1.073×10^-35^ |  |
| ICAR + Gender |  | 0.050 |  | 2.420×10^-52^ |  | 4.598×10^-51^ |  | 288.092 |  | 1.042 |  |
| ICAR |  | 0.050 |  | 2.257×10^-52^ |  | 4.288×10^-51^ |  | 268.709 |  | 0.006 |  |
| Gender |  | 0.050 |  | 5.639×10^-55^ |  | 1.071×10^-53^ |  | 0.671 |  | 0.031 |  |
|  | | | | | | | | | | | |

| Supplementary Table 5: Results of Bayesian analysis of covariance on mean sensitivity for the affective prosody recognition task with group and gender as between-subjects variables and age and International Cognitive Ability Resource intelligence quotient scores as covariates. This analysis was conducted using uniform model priors and default priors on coefficients (*r* scale prior width = 0.5 for fixed effects and 0.354 for covariates). Note: this analysis was performed on the participants with a slope > -0.1.  **Model Comparison** | | | | | | | | | | | |
| --- | --- | --- | --- | --- | --- | --- | --- | --- | --- | --- | --- |
| **Models** | | **P(M)** | | **P(M\|data)** | | **BF_M_** | | **BF_10_** | | **error %** | |
| Null model |  | 0.050 |  | 0.255 |  | 6.492 |  | 1.000 |  |  |  |
| Gender |  | 0.050 |  | 0.464 |  | 16.450 |  | 1.822 |  | 0.011 |  |
| Gender + Group |  | 0.050 |  | 0.053 |  | 1.061 |  | 0.208 |  | 3.206 |  |
| Age + Gender |  | 0.050 |  | 0.049 |  | 0.976 |  | 0.192 |  | 4.944 |  |
| Group |  | 0.050 |  | 0.043 |  | 0.852 |  | 0.169 |  | 0.112 |  |
| Gender + ICAR |  | 0.050 |  | 0.039 |  | 0.780 |  | 0.155 |  | 1.057 |  |
| Age |  | 0.050 |  | 0.026 |  | 0.511 |  | 0.103 |  | 0.002 |  |
| ICAR |  | 0.050 |  | 0.023 |  | 0.456 |  | 0.092 |  | 0.002 |  |
| Age + Gender + Group |  | 0.050 |  | 0.008 |  | 0.157 |  | 0.032 |  | 4.535 |  |
| Age + Group |  | 0.050 |  | 0.008 |  | 0.155 |  | 0.032 |  | 1.143 |  |
| Gender + Group + Gender ✻  Group |  | 0.050 |  | 0.007 |  | 0.128 |  | 0.026 |  | 2.276 |  |
| Age + Gender + ICAR |  | 0.050 |  | 0.007 |  | 0.126 |  | 0.026 |  | 2.363 |  |
| Gender + ICAR + Group |  | 0.050 |  | 0.005 |  | 0.087 |  | 0.018 |  | 3.194 |  |
| ICAR + Group |  | 0.050 |  | 0.004 |  | 0.074 |  | 0.015 |  | 1.175 |  |
| Age + ICAR |  | 0.050 |  | 0.004 |  | 0.071 |  | 0.015 |  | 0.010 |  |
| Gender + ICAR + Group + Gender ✻  Group |  | 0.050 |  | 0.002 |  | 0.046 |  | 0.010 |  | 74.574 |  |
| Age + ICAR + Group |  | 0.050 |  | 0.001 |  | 0.021 |  | 0.004 |  | 2.428 |  |
| Age + Gender + ICAR + Group |  | 0.050 |  | 0.001 |  | 0.021 |  | 0.004 |  | 3.266 |  |
| Age + Gender + Group + Gender ✻  Group |  | 0.050 |  | 0.001 |  | 0.020 |  | 0.004 |  | 4.806 |  |
| Age + Gender + ICAR + Group + Gender ✻  Group |  | 0.050 |  | 1.433×10^-4^ |  | 0.003 |  | 5.629×10^-4^ |  | 4.122 |  |
|  | | | | | | | | | | | |

Supplementary Table 6: Results of Bayesian analysis of covariance on mean reaction time for the affective prosody recognition task with group and gender as between-subjects variables and age and International Cognitive Ability Resource intelligence quotient scores as covariates. This analysis was conducted using uniform model priors and default priors on coefficients (*r* scale prior width = 0.5 for fixed effects and 0.354 for covariates). Note: this analysis was performed on the participants with a slope > -0.1.

| **Model Comparison** | | | | | | | | | | | |
| --- | --- | --- | --- | --- | --- | --- | --- | --- | --- | --- | --- |
| **Models** | | **P(M)** | | **P(M\|data)** | | **BF_M_** | | **BF_10_** | | **error %** | |
| Null model |  | 0.050 |  | 1.776×10^-15^ |  | 3.374×10^-14^ |  | 1.000 |  |  |  |
| Gender + Group + Gender ✻  Group |  | 0.050 |  | 0.301 |  | 8.170 |  | 1.693×10^+14^ |  | 1.968 |  |
| Age + Group |  | 0.050 |  | 0.251 |  | 6.381 |  | 1.416×10^+14^ |  | 2.456 |  |
| Group |  | 0.050 |  | 0.196 |  | 4.627 |  | 1.103×10^+14^ |  | 1.533×10^-20^ |  |
| Age + Gender + Group + Gender ✻  Group |  | 0.050 |  | 0.102 |  | 2.148 |  | 5.721×10^+13^ |  | 1.667 |  |
| Gender + ICAR + Group + Gender ✻  Group |  | 0.050 |  | 0.034 |  | 0.674 |  | 1.930×10^+13^ |  | 19.947 |  |
| Age + ICAR + Group |  | 0.050 |  | 0.033 |  | 0.651 |  | 1.865×10^+13^ |  | 1.446 |  |
| Age + Gender + Group |  | 0.050 |  | 0.025 |  | 0.495 |  | 1.428×10^+13^ |  | 3.000 |  |
| Gender + Group |  | 0.050 |  | 0.022 |  | 0.427 |  | 1.236×10^+13^ |  | 1.500 |  |
| ICAR + Group |  | 0.050 |  | 0.017 |  | 0.325 |  | 9.472×10^+12^ |  | 1.484 |  |
| Age + Gender + ICAR + Group + Gender ✻  Group |  | 0.050 |  | 0.013 |  | 0.256 |  | 7.491×10^+12^ |  | 2.387 |  |
| Age + Gender + ICAR + Group |  | 0.050 |  | 0.004 |  | 0.069 |  | 2.051×10^+12^ |  | 5.325 |  |
| Gender + ICAR + Group |  | 0.050 |  | 0.002 |  | 0.038 |  | 1.114×10^+12^ |  | 3.122 |  |
| Age |  | 0.050 |  | 5.940×10^-10^ |  | 1.129×10^-8^ |  | 334506.710 |  | 0.003 |  |
| Age + Gender |  | 0.050 |  | 7.870×10^-11^ |  | 1.495×10^-9^ |  | 44314.753 |  | 1.198 |  |
| Age + ICAR |  | 0.050 |  | 7.489×10^-11^ |  | 1.423×10^-9^ |  | 42173.785 |  | 0.002 |  |
| Age + Gender + ICAR |  | 0.050 |  | 9.631×10^-12^ |  | 1.830×10^-10^ |  | 5423.193 |  | 1.102 |  |
| Gender |  | 0.050 |  | 2.386×10^-16^ |  | 4.533×10^-15^ |  | 0.134 |  | 0.138 |  |
| ICAR |  | 0.050 |  | 1.707×10^-16^ |  | 3.244×10^-15^ |  | 0.096 |  | 0.002 |  |
| Gender + ICAR |  | 0.050 |  | 2.161×10^-17^ |  | 4.106×10^-16^ |  | 0.012 |  | 1.288 |  |
|  | | | | | | | | | | | |

**Overarching Analyses**

*Correlations.* We conducted a pairwise correlational analysis for each group to investigate the relationships between accuracy (for the facial emotion recognition measures) and sensitivity (for the affective prosody experiment). Only participants who completed all three tasks (autistic N = 294; non autistic N = 276) were included in this analysis (and the logistic regression). Supplementary Figure 4 depicts the relationships among these variables. Alpha was set to .05 but Bonferroni corrected for multiple comparisons. Due to the 6 correlations assessed, this resulted in an alpha of .008.


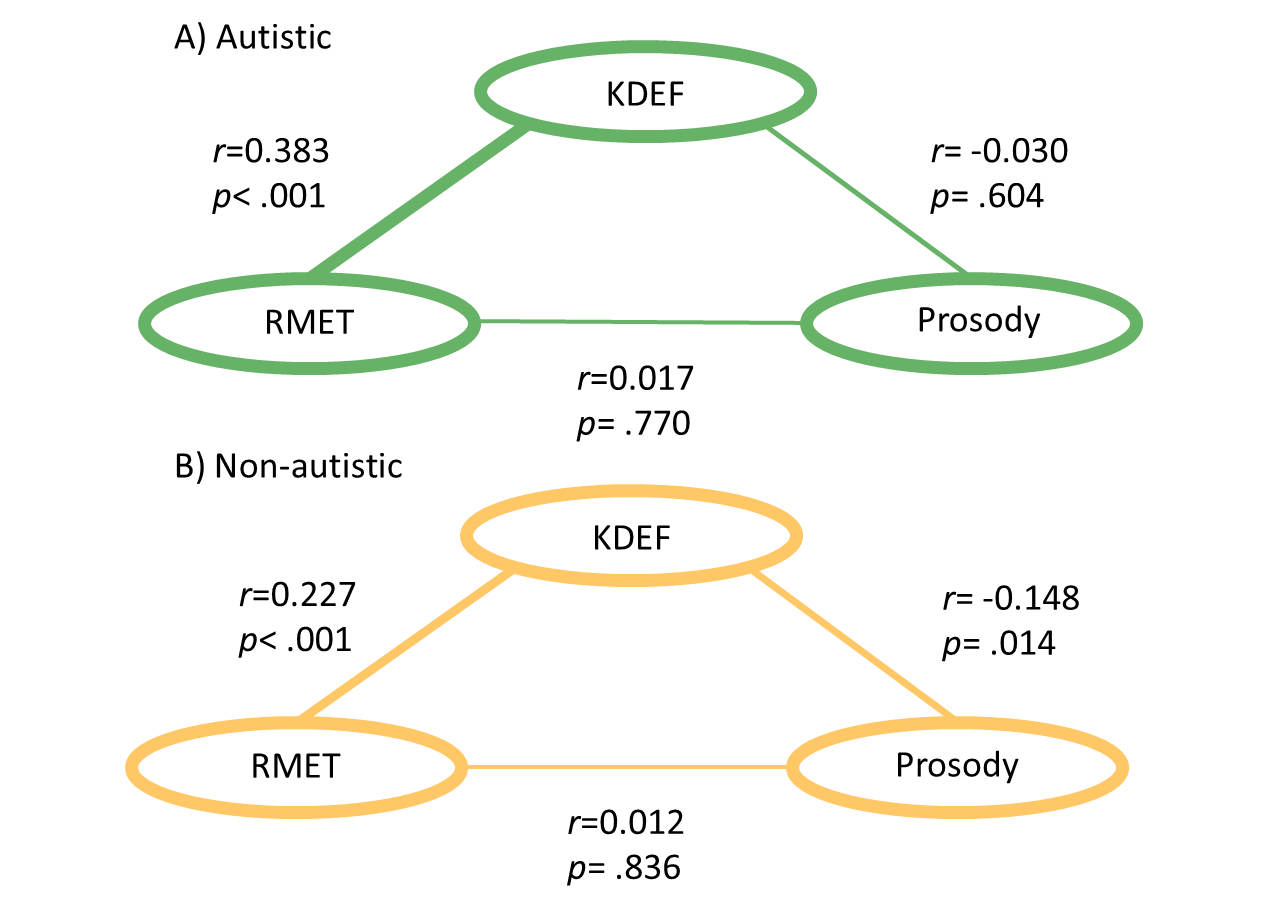


Supplementary Figure 4: A) Correlations between measures for the autistic group. B) Correlations between measures for the non-autistic group. The widths of the lines between nodes scale to the corresponding correlation coefficients.

Analysis of the relationships between measures revealed that both the autistic group (*r*(292) = 0.383, *p* < .001) and the non-autistic group (*r*(274) = 0.227, *p* < .001) showed significant correlations between KDEF and RMET. A two-tailed Fisher r-to-z transformation revealed that the correlation between RMET and KDEF was significantly stronger in the autistic group (*z* = 2.05, *p* = 0.04).

*Logistic regression.* We entered accuracy/sensitivity, RT, and time-out data for all three measures into a logistic regression to assess how predictive of an autism diagnosis each factor was. Supplementary Table 7 illustrates the results of this regression.

Supplementary Table 7: Results of a logistic regression predicting autism diagnosis with accuracy/sensitivity, reaction time, and time-out data from the basic facial emotion recognition task, the complex facial emotion recognition task, and the affective prosody task. Significant predictors are in bold.

| **Model Summary - group** | | | | | | | | | | | | | | | | | | | | | | | | | |  |  |  |
| --- | --- | --- | --- | --- | --- | --- | --- | --- | --- | --- | --- | --- | --- | --- | --- | --- | --- | --- | --- | --- | --- | --- | --- | --- | --- | --- | --- | --- |
| **Model** | | **Deviance** | | **AIC** | | **BIC** | | | **df** | | **Χ²** | | | **p** | | | **McFadden R²** | | **Nagelkerke R²** | | **Tjur R²** | | | **Cox & Snell R²** | |  |  |  |
| H₀ |  | 789.619 |  | 791.619 |  | 795.965 |  | | 569 |  |  | |  |  |  | |  |  |  |  |  | |  |  |  |  |  |  |
| H₁ |  | 642.604 |  | 660.604 |  | 699.715 |  | | 561 |  | 147.015 | |  | < .001 |  | | 0.186 |  | 0.303 |  | 0.236 | |  | 0.227 |  |  |  |  |
|  | | | | | | | | | | | | | | | | | | | | | | | | | |  |  |  |
| **Coefficients** | | | | | | | | | | | | | | | | | | | | | | | | | | | | |
|  | | | | | | | | | | | | | | | | | | | | | | **Wald Test** | | | | | | |
|  | | **Estimate** | | | | | | **Standard Error** | | | | | | | | **z** | | | | | | **Wald Statistic** | | | **df** | | **p** | |
| (Intercept) |  | 4.317 | | |  | | | 1.422 | | | |  | | | | 3.035 | | |  | | | 9.213 | |  | 1 |  | 0.002 |  |
| **kdef_RT** |  | **-0.001** | | |  | | | **0.000** | | | |  | | | | **-4.956** | | |  | | | **24.562** | |  | **1** |  | **< .001** |  |
| kdef_accuracy |  | 0.362 | | |  | | | 1.531 | | | |  | | | | 0.237 | | |  | | | 0.056 | |  | 1 |  | 0.813 |  |
| kdef_out_of_time |  | 0.666 | | |  | | | 19.651 | | | |  | | | | 0.034 | | |  | | | 0.001 | |  | 1 |  | 0.973 |  |
| **rme_RT** |  | **-0.000** | | |  | | | **0.000** | | | |  | | | | **-3.732** | | |  | | | **13.931** | |  | **1** |  | **< .001** |  |
| rme_accuracy |  | 0.232 | | |  | | | 0.932 | | | |  | | | | 0.249 | | |  | | | 0.062 | |  | 1 |  | 0.803 |  |
| rme_out_of_time |  | 1.659 | | |  | | | 6.196 | | | |  | | | | 0.268 | | |  | | | 0.072 | |  | 1 |  | 0.789 |  |
| **psd_RT** |  | **-0.674** | | |  | | | **0.336** | | | |  | | | | **-2.003** | | |  | | | **4.010** | |  | **1** |  | **0.045** |  |
| psd_slope |  | 0.276 | | |  | | | 0.506 | | | |  | | | | 0.547 | | |  | | | 0.299 | |  | 1 |  | 0.585 |  |
|  |  |  | | |  | | |  | | | |  | | | |  | | |  | | |  | |  |  |  |  |  |
|  | | | | | | | | | | | | | | | | | | | | | | | | | | | | |
| *Note.*  group level 'no_autism' coded as class 1.  RT: reaction time  KDEF: Karolinska Directed Emotional Faces  RME: Reading the Mind in the Eyes Test  PSD: affective prosody task | | | | | | | | | | | | | | | | | | | | | | | | | | | | |
|  | | | |  |  |  |  |  |  |  |  |  |  |  |  |  |  |  |  |  |  |  |  |  |  |  |  |  |
| **Performance metrics** | | | | |  |  |  |  |  |  |  |  |  |  |  |  |  |  |  |  |  |  |  |  |  |  |  |  |
|  | | | **Value** | |  |  |  |  |  |  |  |  |  |  |  |  |  |  |  |  |  |  |  |  |  |  |  |  |
| Accuracy | |  | 0.721 |  |  |  |  |  |  |  |  |  |  |  |  |  |  |  |  |  |  |  |  |  |  |  |  |  |
| Sensitivity | |  | 0.721 |  |  |  |  |  |  |  |  |  |  |  |  |  |  |  |  |  |  |  |  |  |  |  |  |  |
| Specificity | |  | 0.721 |  |  |  |  |  |  |  |  |  |  |  |  |  |  |  |  |  |  |  |  |  |  |  |  |  |
|  | | | | |  |  |  |  |  |  |  |  |  |  |  |  |  |  |  |  |  |  |  |  |  |  |  |  |
